# Nanosecond pulsed electric fields induce extracellular release of chromosomal DNA and histone citrullination in neutrophil-differentiated HL-60 cells

**DOI:** 10.1038/s41598-019-44817-9

**Published:** 2019-06-11

**Authors:** Tsubasa Koga, Keiko Morotomi-Yano, Takashi Sakugawa, Hisato Saitoh, Ken-ichi Yano

**Affiliations:** 1 Graduate School of Science and Technology, Kumamoto University, Kumamoto, 860-8555 Japan; 2 Institute of Pulsed Power Science, Kumamoto University, Kumamoto, 860-8555 Japan; 3 Faculty of Advanced Science and Technology (FAST), Kumamoto University, Kumamoto, 860-8555 Japan

**Keywords:** Biophysics, Cell biology

## Abstract

Nanosecond pulsed electric fields (nsPEFs) have gained attention as a novel physical stimulus for life sciences. Although cancer therapy is currently their promising application, nsPEFs have further potential owing to their ability to elicit various cellular responses. This study aimed to explore stimulatory actions of nsPEFs, and we used HL-60 cells that were differentiated into neutrophils under cultured conditions. Exposure of neutrophil-differentiated HL-60 cells to nsPEFs led to the extracellular release of chromosomal DNA, which appears to be equivalent to neutrophil extracellular traps (NETs) that serve as a host defense mechanism against pathogens. Fluorometric measurement of extracellular DNA showed that DNA extrusion was rapidly induced after nsPEF exposure and increased over time. Western blot analysis demonstrated that nsPEFs induced histone citrullination that is the hydrolytic conversion of arginine to citrulline on histones and facilitates chromatin decondensation. DNA extrusion and histone citrullination by nsPEFs were cell type-specific and Ca^2+^-dependent events. Taken together, these observations suggest that nsPEFs drive the mechanism for neutrophil-specific immune response without infection, highlighting a novel aspect of nsPEFs as a physical stimulus.

## Introduction

Nanosecond pulsed electric fields (nsPEFs) are increasingly recognized as a novel physical means by which unique biological effects can be achieved. Exposure of cells to nsPEFs generates small membrane pores, namely nanopores, which have channel-like properties^1^. Consistent with nanopore formation, nsPEFs elicit the influx of extracellular Ca^2+ ^^2–4^ and the permeation of small fluorescent dyes, such as YO-PRO1^5^. In addition to nanopore-mediated membrane permeabilization, nsPEFs induce various cellular responses in a Ca^2+^-dependent and -independent manner. The exposure of cells to nsPEFs causes the rapid externalization of phosphatidylserine on the cell membrane^5^, which can be accounted for, in part, by the activation of TMEM16F, a Ca^2+^-dependent phospholipid scramblase^6^. nsPEFs have been reported to activate multiple intracellular signaling pathways, including the MAPK, AMPK and phosphoinositide pathways^7–10^, the activation of some of which is dependent on the presence of extracellular Ca^2+ ^^7^. Relatively intense nsPEFs act as a novel form of cellular stress and induce eIF2α phosphorylation and 4E-BP1 dephosphorylation, both of which lead to translational suppression^11^. nsPEF-induced stress responses are largely Ca^2+^-independent, suggesting that nsPEFs directly affect intracellular components.

In addition to their unique effect on the cell membrane and intracellular signaling, nsPEFs attract particular attention owing to their ability to induce cell death. Apoptotic and non-apoptotic cell death can be induced by nsPEFs in a cell type-dependent manner. For example, nsPEFs induce apoptosis in HL-60 and Jurkat cells irrespective of the presence of extracellular Ca^2+ ^^12–14^. On the other hand, HeLa cells exposed to nsPEFs undergo non-apoptotic cell death in a Ca^2+^-dependent manner^15^. Non-apoptotic cell death by nsPEFs is associated with the formation of gross protein crosslinking mediated by the Ca^2+^-dependent enzyme transglutaminase 2^16^. Importantly, the exposure of cancer cells to nsPEFs leads to the release of a distinct set of intracellular proteins, which represent damage-associated molecular patterns (DAMPs)^17,18^. The release of DAMPs from dying cells can initiate both innate and adaptive immune responses *in vivo*. Thus, nsPEFs hold great promise for cancer therapy because of the synergistic effects of cytotoxicity and cancer-derived DAMPs.

While cytotoxic effects of nsPEFs on tumor-derived cells in relation to cancer therapy have received considerable attention, effects of nsPEFs on other cell systems have been also extensively studied, such as permeabilization of human eosinophils^19^ and the activation of cardiac myocytes^20,21^, hippocampal neurons^22,23^, platelets^24^, and chromaffin cells^25^. In this study, we investigated stimulatory actions of nsPEFs on human HL-60 cells that were differentiated into neutrophils under cultured conditions. Neutrophils are the most abundant type of white blood cells and play a critical role in innate immunity^26^. In the human body, neutrophils differentiate from hematopoietic stem cells in the bone marrow and have a short life cycle in blood stream^26^. Neutrophils have several mechanisms to combat infection, such as phagocytosis. In response to specific stimuli, a subset of neutrophils undergoes a unique immune response: they release their chromosomal DNA from the nucleus into the extracellular space. This extruded DNA from the neutrophil nucleus is called neutrophil extracellular traps (NETs). Because the extracellular release of chromosomal DNA ultimately leads to cell death, which is called NETosis, NET formation is also regarded as an active mechanism for cell death^27^. NETs are mainly composed of decondensed chromatin fibers and tend to form aggregates that are detectable using a fluorescent DNA dye. NETs associate with antimicrobial proteins to trap and kill microbes. Moreover, NETs can facilitate phagocytosis by other neutrophils or macrophages^28^ and blood coagulation^29^. The cellular mechanism of DNA extrusion for antimicrobial defense has been found in animals, plants^30^, insects^31^, and even the social amoeba *Dictyostelium discoideum*^32^, indicating that the DNA-based defense system has an ancient evolutionary origin.

In the nucleus, DNA is tightly associated with histone octamers to form nucleosomes and is further assembled into highly compacted chromatin. Each nucleosomal histone contains multiple basic amino acids, such as arginine, and the strong positive charges of histones result in tight electrostatic binding to DNA and consequent chromatin compaction. NETs are extracellular chromatin fibers in a decondensed form and contain post-translationally modified histones with the non-conventional amino acid citrulline. Prior to the extrusion of chromosomal DNA into the extracellular space, arginine residues in histones are hydrolyzed, resulting in the conversion of arginine to citrulline. This process is called citrullination and reduces the positive charges of histones, which promotes chromatin decondensation and DNA extrusion from the nucleus^33^. In neutrophils, histone citrullination is primarily catalyzed by peptidylarginine deiminase 4 (PAD4), a Ca^2+^-dependent enzyme^34^. Histone citrullination is currently regarded as a molecular signature of NET formation.

In this study, we applied nsPEFs to neutrophil-differentiated HL-60 cells and observed the extracellular release of chromosomal DNA and concomitant histone citrullination. The presence of extracellular Ca^2+^ was essential for the induction of these events. Our findings suggest that nsPEFs drive the mechanism for NET formation without infection. This study extends the potential of nsPEFs as a physical stimulus and provides possible implications for clinical applications of nsPEFs.

## Results

### Differentiation of HL-60 cells into neutrophils

**Figure 1:**
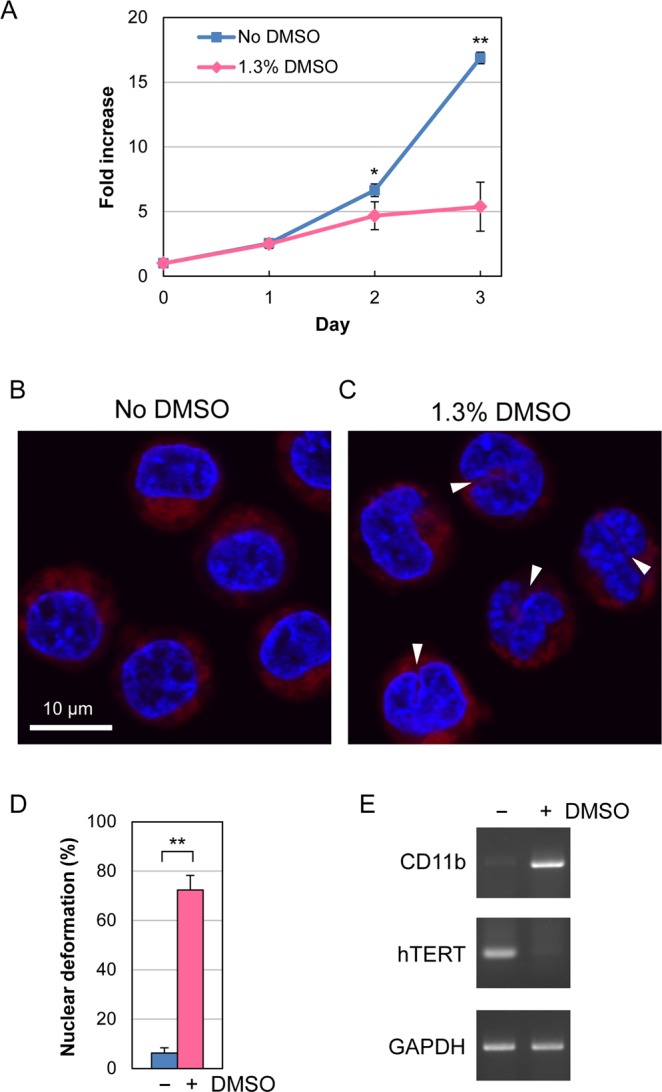
Differentiation of HL-60 cells into neutrophils. (**A**) Growth of HL-60 in the presence and absence of 1.3% DMSO. Average fold increases in cell number with SD were calculated from 5 independent experiments (*p < 0.05; **p < 0.01). (**B**,**C**) Fluorescence microscopy of HL-60 cells. Cells were cultured in the absence (**B**) and presence (**C**) of 1.3% DMSO for 3 days and stained with Hoechst 33342 for DNA (blue) and MitoRed for mitochondria (red). White arrow heads indicate the invagination of the nuclear rim. (**D**) Quantification of nuclear deformation. Fluorescence microscopy was performed as in (**B**,**C**), and cells exhibiting the invagination of the nuclear rim at one or more sites were scored as nuclear lobulated cells. At least 200 cells were inspected by microscopy in each experiment. Average values with SD were calculated from 6 independent experiments (**p < 0.01). (**E**) RT-PCR analysis of differentiation-related gene expression. HL-60 cells were cultured in the presence and absence of 1.3% DMSO for 3 days and subjected to RT-PCR analysis. CD11b is known to be expressed in neutrophil-differentiated cells but not in undifferentiated ones. Conversely, expression of hTERT is attenuated during differentiation. The GAPDH gene is constitutively expressed in both cell types. Uncropped images are shown in Supplementary Fig. S4.

In this study, we used human acute myeloid leukemia HL-60 cells as a model for investigating the effects of nsPEFs on immune cells. As reported previously, HL-60 cells can be differentiated into neutrophil cells in the presence of low concentrations of dimethyl sulfoxide (DMSO)^35^. We thus cultured HL-60 cells in the presence and absence of 1.3% DMSO for 3 days and analyzed the characteristics of these cells. First, we measured cell proliferation and observed that the growth rate of DMSO-treated cells slowed down in comparison to untreated cells, presumably reflecting the progress of neutrophil differentiation in DMSO-treated cells (Fig. 1A). Next, we analyzed nuclear morphology because neutrophils are known to exhibit a morphological change of the nucleus, known as nuclear lobulation, in which the nuclear rim is invaginated. Using fluorescence microscopy, we observed nuclear lobulation in DMSO-treated cells, but not in untreated cells (Fig. 1B,C). The percentage of DMSO-treated cells exhibiting invagination of the nuclear rim reached more than 70% (Fig. 1D). Finally, we examined the gene expression characteristic of neutrophil-differentiated and undifferentiated cells. CD11b belongs to the integrin family, and its expression is virtually absent in undifferentiated HL-60 cells but is highly activated in neutrophil-differentiated cells^36^. Conversely, the telomerase reverse transcriptase hTERT gene is constitutively expressed in undifferentiated HL-60 cells, and its transcription is down-regulated during differentiation^37^. RT-PCR analysis showed the activation of CD11b expression and the suppression of hTERT expression in DMSO-treated cells (Fig. 1E). Taken together, these observations indicate that DMSO-treated cells differentiated into neutrophils. We therefore used DMSO-treated HL-60 cells as neutrophil-differentiated cells to study the effect of nsPEFs.

### Microscopic observation of extracellular DNA

**Figure 2:**
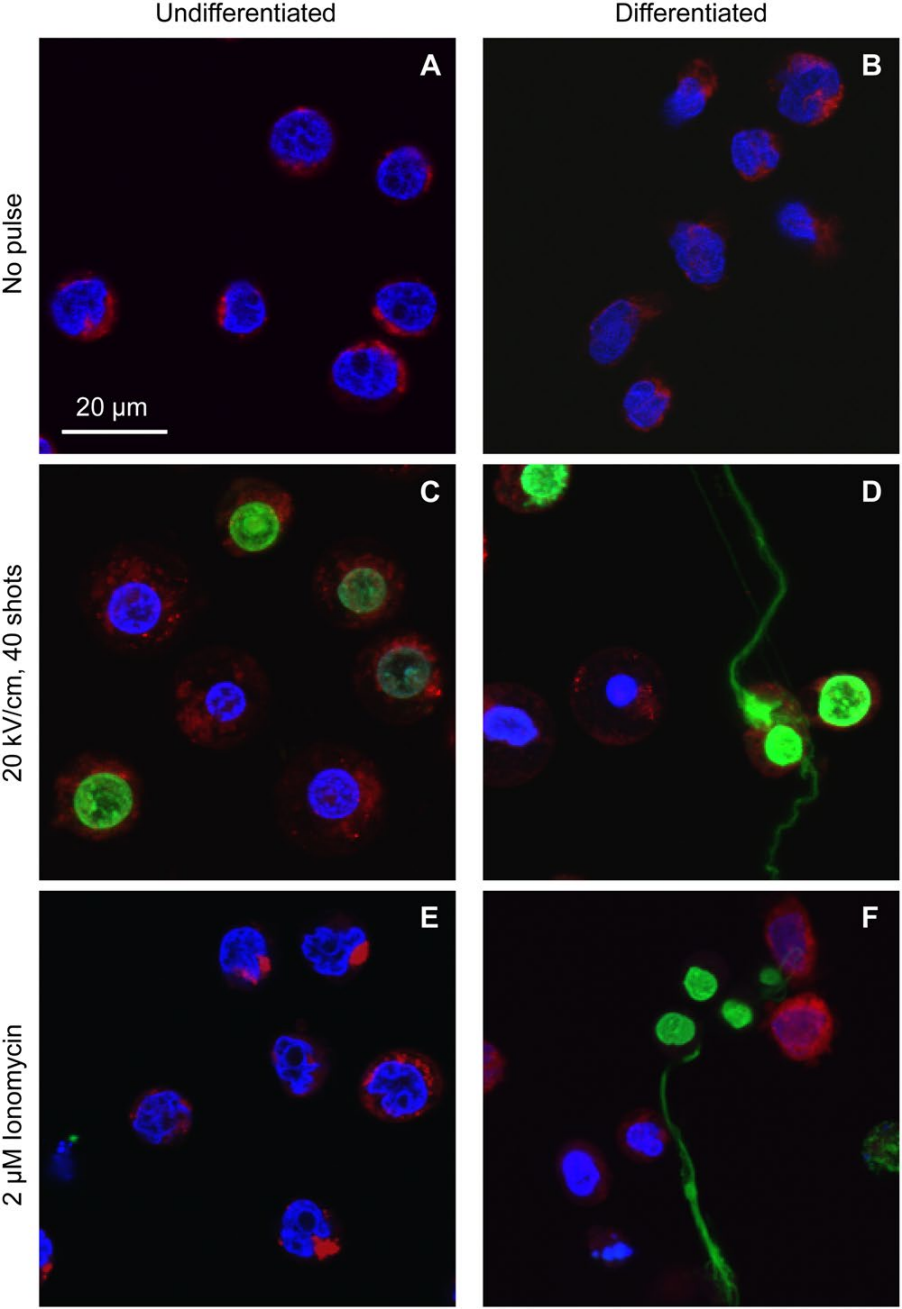
Microscopic observation of extracellular DNA. Differentiated and undifferentiated HL-60 cells were exposed to 40 shots of 20 kV/cm nsPEFs or treated with 2 µM ionomycin. After 1 h incubation at 37 °C, cells were stained with SYTOX Green, Hoechst 33342 and MitoRed. Typical images are represented.

Next, we performed fluorescence microscopy to examine whether nsPEFs would induce the extracellular release of chromosomal DNA. Cells were exposed to 40 shots of 80 nsPEFs at 20 kV/cm and incubated at 37 °C for 1 h. Subsequently, cells were stained with three fluorescent dyes. Hoechst 33342 and MitoRed are cell-permeable dyes that stain DNA and mitochondria, respectively. SYTOX Green also stains DNA but does not permeate through intact cell membranes. Without nsPEF exposure, neither undifferentiated nor differentiated cells were stained with SYTOX Green (Fig. 2A,B). When undifferentiated cells were exposed to nsPEFs, the nuclei were stained green, indicating that nsPEFs permeabilized the cell membrane and allowed the entry of SYTOX Green into the cells, as is the case with YO-PRO1^5^ (Fig. 2C). When differentiated cells were exposed to nsPEFs, in addition to nuclear staining, filamentous green staining was detected in the extracellular space (Fig. 2D). When we treated the cells with the calcium ionophore ionomycin, which is known to induce NET formation^38^, we observed similar extracellular filamentous green staining in differentiated cells, but not in undifferentiated cells (Fig. 2E,F). Based on these observations, we speculated that neutrophil-differentiated HL-60 cells released their chromosomal DNA into the extracellular space in response to nsPEFs.

### Analysis of extracellular DNA by agarose gel electrophoresis

**Figure 3:**
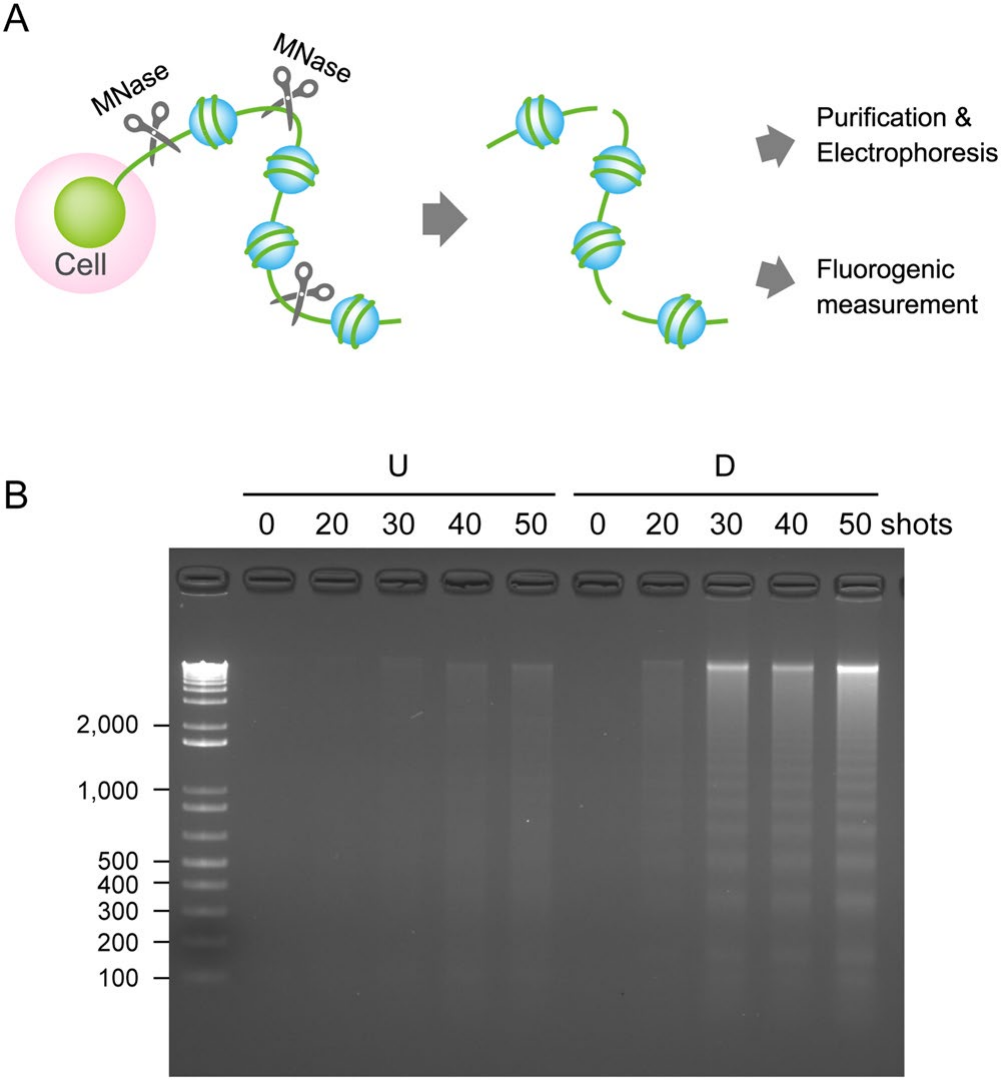
Analysis of extracellular DNA by agarose gel electrophoresis. (**A**) Schematic representation of MNase treatment for liberation of extruded DNA from cells. MNase digests extracellular chromosomal DNA at its linker regions among nucleosomes, leading to the liberation of extracellular chromosomal DNA from their originating cells. Liberated DNA was separated from cells by brief centrifugation and in turn subjected to either DNA purification followed by agarose gel electrophoresis (**B**) or direct fluorometric measurement with a SYTOX Green dye (Fig. 4). (**B**) Analysis of MNase-treated extracellular DNA by agarose gel electrophoresis. Differentiated (D) and undifferentiated cells (U) were exposed to the indicated shot numbers of 20 kV/cm nsPEFs. After 30 min incubation at 37 °C, the cell suspension was digested with MNase, and extracellular DNA was separated from the cells by centrifugation. DNA was purified by proteinase K digestion followed by ethanol precipitation, and analyzed by agarose gel electrophoresis. An uncropped image is shown in Supplementary Fig. S5.

To confirm that extracellular filamentous staining represents chromosomal DNA, we attempted to separate the extracellular DNA from the cells and analyze this by agarose gel electrophoresis. Because NET DNA is generally connected with their originating neutrophils, the cell suspension exposed to nsPEFs was briefly treated with micrococcal nuclease (MNase), which preferentially cuts DNA strands at the linker regions of nucleosomes (Fig. 3A). Liberated nucleosomal DNA fragments were separated from the cells by centrifugation, then purified by proteinase K digestion followed by ethanol precipitation, and finally analyzed using agarose gel electrophoresis. As shown in Fig. 3B, we observed DNA laddering at approximately 200 bp intervals in the suspensions of nsPEF-exposed differentiated cells. The DNA ladder formation was not detectable without exposure to nsPEFs (Fig. 3B). These results indicate that extracellular filamentous staining in Fig. 2D represents chromosomal DNA, and we supposed that the extracellular release of chromosomal DNA from differentiated HL-60 cells is equivalent to the formation of NETs in neutrophils. When undifferentiated cells were used, intense nsPEFs (e.g. 50 shots) yielded faint DNA signals. Because fluorescence microscopy did not show extracellular filamentous staining in nsPEF-exposed undifferentiated cells (Fig. 2C), we speculated that intense nsPEF treatment disrupted the cell membrane and caused intracellular entry of MNase, such that these faint signals do not represent the extracellular release of chromosomal DNA.

### Fluorometric measurement of DNA extrusion

**Figure 4:**
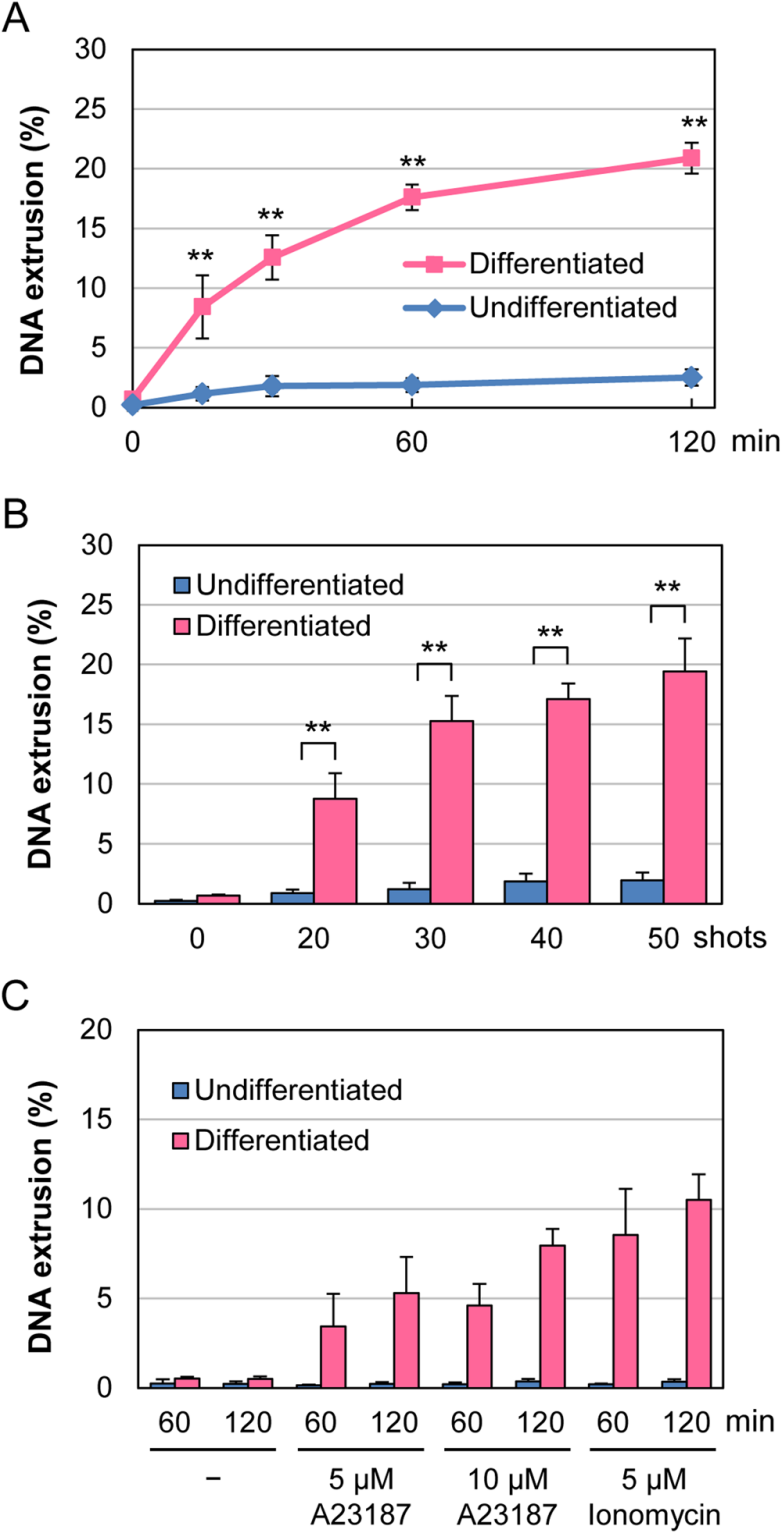
Fluorometric measurement of DNA extrusion. (**A**) Time-course of DNA extrusion after nsPEF exposure. Differentiated and undifferentiated HL-60 cells were exposed to 40 shots of 20 kV/cm nsPEFs and incubated at 37 °C for the indicated time periods. DNA extrusion was expressed as a ratio of extracellular DNA to total DNA. Average values with SD were calculated from 5 independent experiments (**p < 0.01 between differentiated and undifferentiated cells). (**B**) Effects of different shot numbers of nsPEFs on DNA extrusion. The indicated numbers of shots of 20 kV/cm nsPEFs were applied to cells. After 1 h incubation at 37 °C, the cells were analyzed as described in A (n = 5; **p < 0.01 between differentiated and undifferentiated cells). (**C**) Induction of DNA extrusion by the calcium ionophores A23187 and ionomycin. Differentiated and undifferentiated HL-60 cells were treated with the indicated concentrations of calcium ionophores. After the indicated periods of incubation at 37 °C, the cells were analyzed as described in A (n = 5).

Next, we attempted to quantitatively assess the extracellular release of chromosomal DNA. Extracellular DNA was separated from cells by gentle MNase digestion followed by centrifugation and then stained with SYTOX Green (Fig. 3A). In parallel, total DNA was prepared and mixed with SYTOX Green. Fluorescence of extracellular and total DNA was measured using a fluorescence plate reader, and DNA extrusion from the nucleus was expressed as a ratio of extracellular DNA to total DNA. Using this method, we observed marked increases in DNA extrusion in differentiated cells after nsPEF exposure (Fig. 4). Time-course analysis showed that DNA extrusion was rapidly induced after nsPEF exposure and increased over time (Fig. 4A). As the shot numbers of nsPEFs increased, DNA extrusion also increased (Fig. 4B). We did not observe such increases in DNA extrusion in undifferentiated cells (Fig. 4A,B).

Because Ca^2+^ ionophores are known to induce NET formation^38^, we next quantitatively assessed DNA extrusion induced by A23187 and ionomycin, both of which are Ca^2+^ ionophores. As shown in Fig. 4C, these Ca^2+^ ionophores induced DNA extrusion in neutrophil-differentiated cells but not in undifferentiated ones. Compared to nsPEFs (Fig. 4B), these compounds yielded DNA extrusion to a lesser extent.

### Induction of histone citrullination by nsPEFs

**Figure 5:**
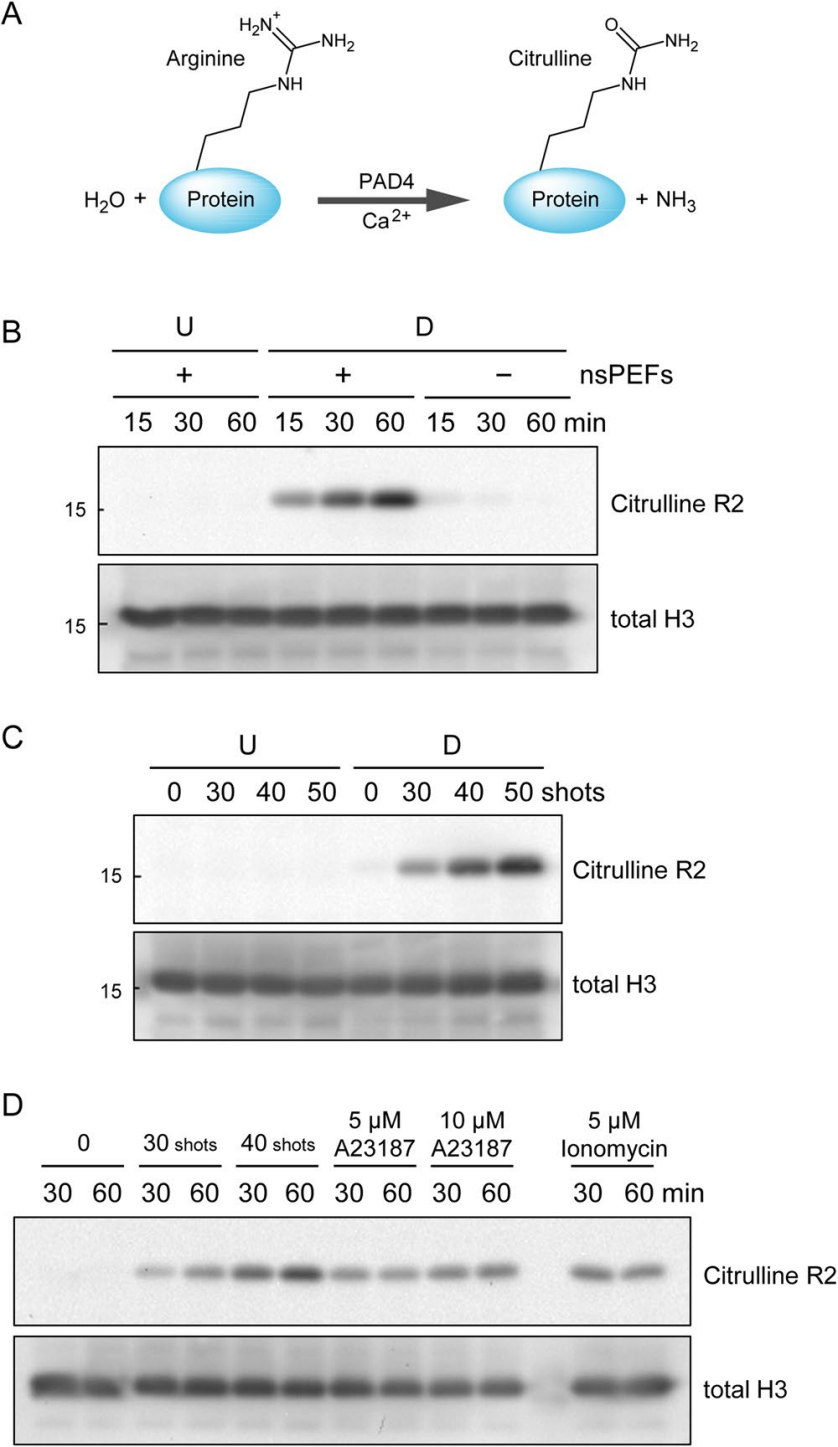
nsPEF-induced histone citrullination. (**A**) Schematic representation of citrullination of an arginine residue. PAD4 catalyzes hydrolytic conversion of arginine to citrulline in the presence of Ca^2+^. (**B**) Time-course of histone H3 citrullination after nsPEF exposure. Differentiated (D) and undifferentiated (U) HL-60 cells were treated with (+) and without (−) 40 shots of 20 kV/cm nsPEFs. After the indicated periods of incubation at 37 °C, the cells were collected and subsequently subjected to western blot analysis using an antibody against citrullinated histone H3 at R2. Total histone H3 levels were shown in the lower panel as a loading control. Original western blot images are shown in Supplementary Fig. S6. (**C**) Effects of different shot numbers of nsPEFs on histone citrullination. Indicated shot numbers of 20 kV/cm nsPEFs were applied to undifferentiated (U) and differentiated (D) HL-60 cells. Cells were incubated at 37 °C for 30 min and in turn subjected to western blot analysis as described in B. Original western blot images are shown in Supplementary Fig. S7. (**D**) Induction of histone citrullination by calcium ionophores. Differentiated HL-60 cells were treated with either 20 kV/cm nsPEFs or calcium ionophores as indicated in the panel. After the indicated periods of incubation at 37 °C, cells were subjected to western blot analysis as described in B. Original western blot images are shown in Supplementary Fig. S8.

The DNA in the nucleus forms highly condensed structures, which should be loosened prior to NET formation. Citrullination (Fig. 5A) weakens the histone-DNA interactions and facilitates the decondensation of packed chromatin and the release of chromosomal DNA. We investigated whether nsPEFs induce histone citrullination. Western blot analysis showed that nsPEF exposure led to the induction of histone citrullination in neutrophil-differentiated cells (Fig. 5B). When the shot numbers of nsPEFs increased, histone citrullination was enhanced (Fig. 5C). Without nsPEF exposure, histone citrullination was barely detectable in differentiated cells (Fig. 5B,C). In undifferentiated cells, nsPEFs did not induce histone citrullination (Fig. 5B,C). When neutrophil-differentiated cells were treated with the Ca^2+^ ionophores A23187 or ionomycin, histone citrullination was induced (Fig. 5D). We confirmed the absence of histone citrullination in undifferentiated cells treated with A23187 (Supplementary Fig. S1). We observed that nsPEF exposure caused stronger signals of histone citrullination than treatment with Ca^2+^ ionophores (Fig. 5D). This observation was in line with the results of the quantitative assessment of DNA extrusion, in which nsPEFs (Fig. 4B) yielded more DNA extrusion than Ca^2+^ ionophores (Fig. 4C).

### Ca^2+^-dependent DNA extrusion and histone citrullination by nsPEFs

**Figure 6:**
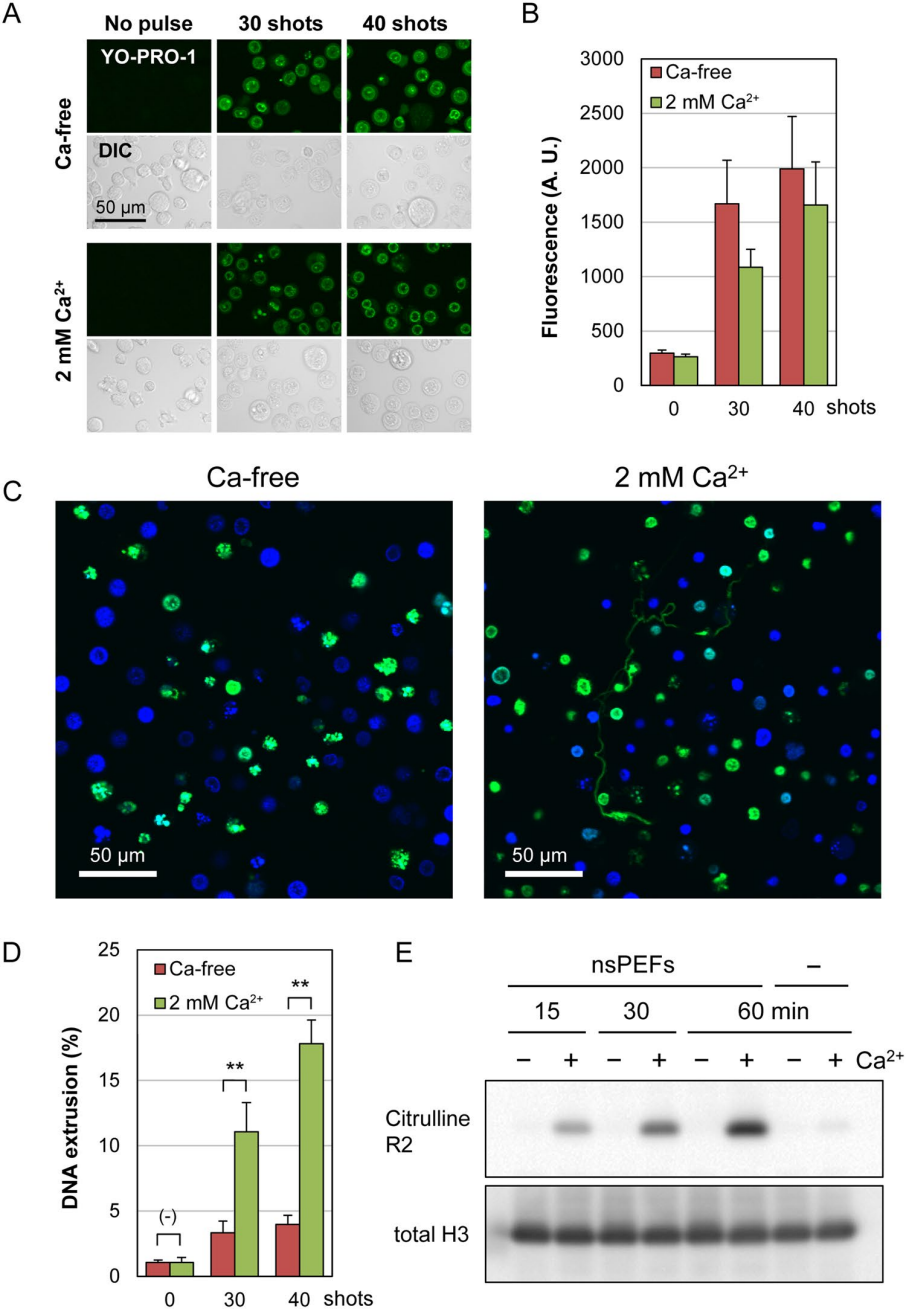
Calcium-dependent induction of DNA extrusion and histone citrullination by nsPEFs. (**A**) YO-PRO-1 staining of neutrophil-differentiated cells after nsPEF exposure in the presence and absence of 2 mM Ca^2+^. Indicated shot numbers of 20 kV/cm nsPEFs were applied to neutrophil-differentiated HL-60 cells in the presence of 1 µM YO-PRO-1. At 5 min after nsPEF exposure, green fluorescent and differential interference contrast (DIC) images were captured. (**B**) Quantification of YO-PRO-1 staining of nsPEF-exposed cells in the presence and absence of Ca^2+^. Cells were exposed to nsPEFs and stained with YO-PRO-1 as described in (**A**) and fluorescent images were acquired using the identical settings of microscopy. YO-PRO-1 staining of individual cells was quantified using FLUOVIEW software and expressed in arbitrary units (A.U.). Average values with SD were calculated from 120 cells in each assay. (**C**) Microscopic analysis of extracellular DNA in the presence and absence of extracellular Ca^2+^. Differentiated HL-60 cells were suspended in either Ca^2+^-containing (2 mM) or Ca^2+^-free HBS and exposed to 40 shots of 20 kV/cm nsPEFs. After 1 h incubation at 37 °C, cells were stained with SYTOX Green and Hoechst 33342. (**D**) Fluorometric analysis of DNA extrusion in the presence and absence of extracellular Ca^2+^. Differentiated HL-60 cells were exposed to 40 shots of 20 kV/cm nsPEFs in the presence and absence of 2 mM Ca^2+^. After 1 h incubation at 37 °C, fluorometric analysis of DNA extrusion was performed as described in Fig. 4. The Ca^2+^-free cell suspension was supplemented with CaCl_2_ solution to yield 2 mM Ca^2+^ and was subjected to MNase treatment followed by fluorometric analysis. Average values with SD were calculated from 5 independent experiments (**p < 0.01 between Ca^2+^-containing and Ca^2+^-free samples). (**E**) Western blot analysis of Ca^2+^-dependent histone H3 citrullination. Differentiated HL-60 cells were suspended in either Ca^2+^-containing (2 mM, +) or Ca^2+^-free (−) HBS and treated with 40 shots of 20 kV/cm nsPEFs. Cells were collected after the indicated time periods and subjected to western blot analysis of citrullinated histone H3. Original western blot images are shown in Supplementary Fig. S9.

Previous studies have shown that nsPEFs induce the influx of Ca^2+^ through the nanopores of the cell membrane^2–4^. To test the relationship between Ca^2+^ influx and DNA extrusion in nsPEF-exposed cells, neutrophil-differentiated cells were suspended in either Ca^2+^-containing or Ca^2+^-free buffer and exposed to nsPEFs. First, we analyzed the influence of Ca^2+^ on nsPEF-induced membrane damages by YO-PRO-1 staining. Under a fluorescent microscope, nearly all cells were stained with YO-PRO-1, irrespective of the presence or absence of calcium (Fig. 6A). We next measured the fluorescence intensity of individual cells and found that cells exhibited slightly stronger fluorescent staining in the absence of calcium (Fig. 6B), indicating that the absence of calcium may cause more membrane damage. This observation was in accordance with previous studies that reported attenuation of YO-PRO-1 incorporation into BPAE cells and CHO-K1 cells in the presence of Ca^2+ ^^39,40^. Next, we carried out fluorescence microscopy of nsPEF-exposed differentiated cells and observed filamentous staining with SYTOX Green in the extracellular space in Ca^2+^-containing but not in Ca^2+^-free cell suspensions (Fig. 6C). Fluorometric measurement of extracellular DNA confirmed that nsPEF-induced DNA extrusion was significantly suppressed in the absence of extracellular Ca^2+^ (Fig. 6D). Furthermore, western blot analysis showed that nsPEF-induced histone citrullination was totally dependent on the presence of extracellular Ca^2+^ and was nearly absent in Ca^2+^-free cell suspension (Fig. 6E). These results suggest that the influx of extracellular Ca^2+^ is a critical factor for nsPEF-induced DNA extrusion and histone citrullination.

### Cell type-dependent DNA extrusion and histone citrullination by nsPEFs

**Figure 7:**
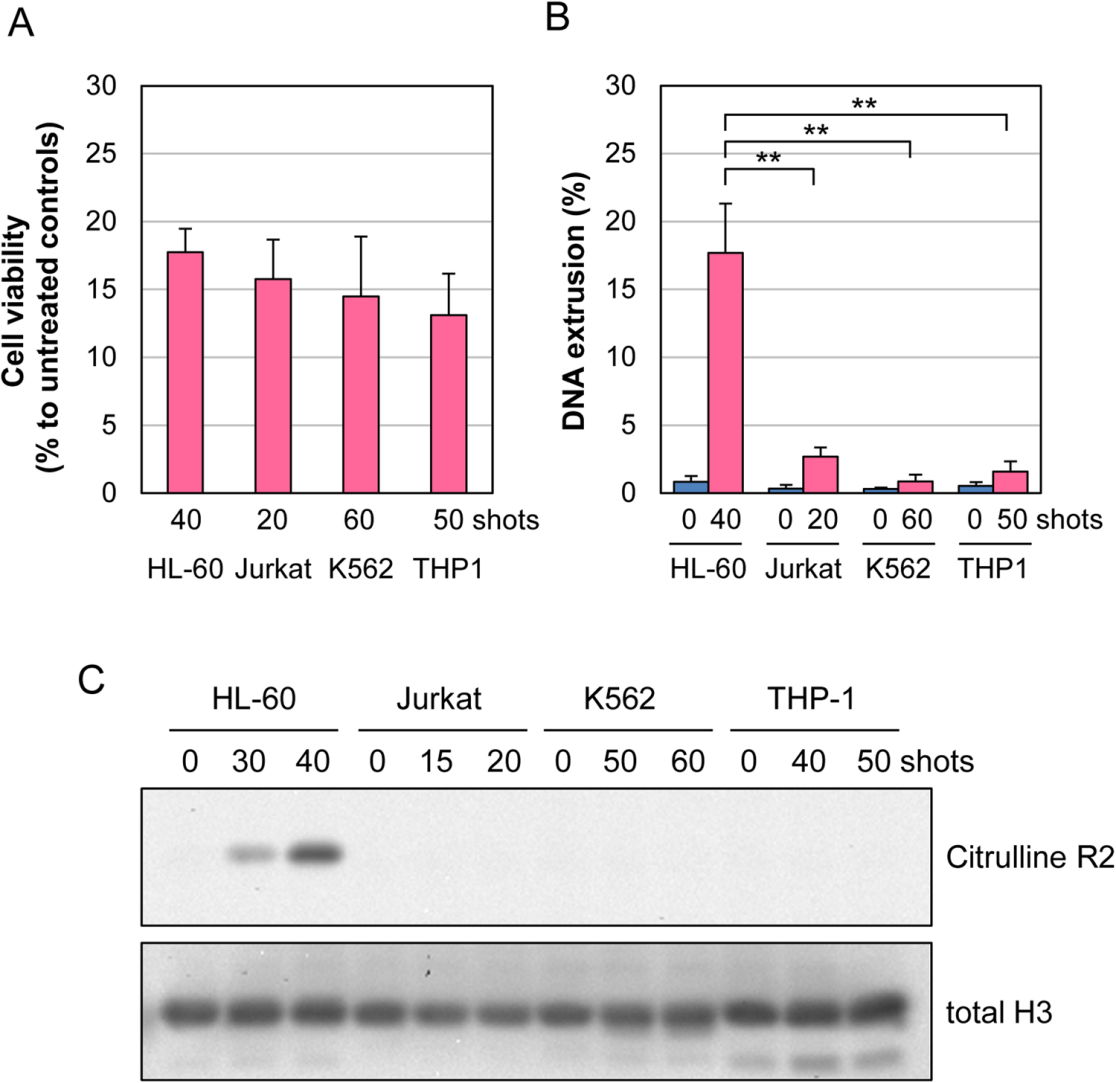
Cell type-dependent induction of DNA extrusion and histone citrullination by nsPEFs. (**A**) Cell viability analysis. Indicated numbers of shots of 20 kV/cm nsPEFs were applied to each cell type, and cell viability was measured at 6 hr. Average values with SD were calculated from 5 independent experiments. (**B**) Measurement of DNA extrusion after nsPEF exposure. Indicated numbers of shots of 20 kV/cm nsPEFs were applied to each cell type, and DNA extrusion was measured at 1 hr. Average values with SD were calculated from 5 independent experiments (**p < 0.01 between neutrophil-differentiated HL-60 cells and other cell types). (**C**) Western blot analysis of histone H3 citrullination after nsPEF exposure. Indicated numbers of shots of 20 kV/cm nsPEFs were applied to each cell type. After incubation at 37 °C for 30 min, western blot analysis was performed as described in Fig. 5. Original western blot images are shown in Supplementary Fig. S10.

To investigate the cell-type specificity of DNA extrusion and histone citrullination by nsPEFs, we analyzed the effects of nsPEFs on other leukemic cell lines, Jurkat, K562 and THP-1. Because previous studies have demonstrated that different cell lines exhibit different sensitivities to nsPEFs^41–47^, we determined nsPEF conditions for each cell type that yielded reduced cell viability to similar extents (Fig. 7A). Using these nsPEF conditions for each cell line, we observed a marked increase in DNA extrusion in differentiated HL-60 cells, but not in other cell types (Fig. 7B). Although slight increases in fluorescent signals were detectable in Jurkat, K562, THP-1 cells after nsPEF exposure, we speculate that MNase entered the severely damaged cells and was able to reach the nucleus. We next performed western blot analysis of the cell lines exposed to respective conditions of nsPEFs and observed that histone citrullination was detectable only in neutrophil-differentiated HL-60 cells (Fig. 7C). Finally, we used the same nsPEF conditions for all cell types and confirmed that DNA extrusion (Supplementary Fig. S2) and histone citrullination (Supplementary Fig. S3) were induced in neutrophil-differentiated HL-60 cells but not in other cell types. Collectively, these results indicate that nsPEFs induce DNA extrusion and histone citrullination in a cell type-dependent manner.

## Discussion

nsPEFs are widely accepted as a novel physical means in life sciences. In particular, nsPEFs have attracted attention as an effective therapeutic modality for solid tumors. In the current study, we explored the usage of nsPEFs for cell stimulation and chose neutrophil-differentiated HL-60 cells as an experimental model. We found that nsPEFs induce the extracellular release of chromosomal DNA and histone citrullination in neutrophil-differentiated HL-60 cells but not in undifferentiated cells or other cell lines. The presence of extracellular Ca^2+^ was essential for these events, highlighting the critical importance of Ca^2+^ influx. These observations suggest that nsPEFs drive the mechanism for NET formation without infection. Our current study is of importance as an attempt to expand the potential of nsPEF use for cell stimulation.

For the analysis of DNA extrusion by nsPEFs, we employed two experimental approaches. First, we used fluorescence microscopy to inspect the presence of extracellular DNA and observed filamentous objects stained with SYTOX Green in the extracellular space. NETs are known to consist of highly decondensed chromatin fibers, which are generally too thin to observe under a microscope. Because NETs tend to form aggregates^48,49^, we speculated that a portion of the extruded DNA formed aggregates to become microscopically visible. This idea further suggests that visible staining under a microscope does not necessarily represent the whole range of extracellularly released DNA. For this reason, we attempted the fluorometric measurement of extracellular DNA that was separated from their originating cells by MNase treatment followed by centrifugation. Total DNA was also measured in parallel, and DNA extrusion was expressed as a ratio of extracellular DNA to total DNA. In most previous studies on NET formation, the quantitative evaluation of NET formation relied on the staining of cells with SYTOX Green. It is widely accepted that SYTOX Green-positive cells can be regarded as having undergone NET formation, because NET formation is accompanied with the rupture of the cell membrane, allowing the entry of SYTOX Green into the cells. In the case of nsPEFs, however, such a conventional staining method is not applicable, because exposure of cells to nsPEFs causes permeation of SYTOX Green across the cell membrane, irrespective of NET formation. Our method for the assessment of extracellular DNA circumvented this point, serving as a valuable complement to the conventional methods relying on cell stain. Thus, this method could be useful for other studies on NET formation.

In this study, we demonstrated the Ca^2+^-dependent induction of DNA extrusion and histone citrullination by nsPEFs in neutrophil-differentiated HL-60 cells. These observations serve as a basis for further investigation of the molecular mechanisms by which nsPEFs act on neutrophil-differentiated cells. Although the induction of NET formation generally results in the same outcome, i.e. the extracellular release of chromosomal DNA, multiple mechanisms could drive this process^50–52^. For example, reactive oxygen species (ROS) participate in NET formation in different ways. In neutrophils, various stimuli cause the activation of NADPH oxidases (NOX) that produce ROS. NOX-mediated ROS production has been repeatedly demonstrated to be critical for the induction of NET formation by microbes and the mitogen phorbol 12-myristate 13-acetate (PMA)^50^. Several studies reported NOX-independent NET formation, in which ROS is produced through metabolic activities in mitochondria^38^. Furthermore, ROS-independent NET formation was also described^53^. It is worth noting that previous studies have demonstrated nsPEF-induced ROS production through both electrochemical and biological processes^54^ and its suppression by a Ca^2+^-chelating agent^55^. It is therefore worthy of further study on the requirement of ROS for nsPEF-induced DNA extrusion as well as the mode of ROS production with respect to neutrophil differentiation and Ca^2+^ dependency.

In addition to ROS, various protein kinases participate in the control of NET induction. PMA-induced NET formation is accompanied by the activation of multiple protein kinases, including PKC, MEK, and ERK, where the inhibition of one of these kinases significantly suppresses NET formation^56^. JNK plays a critical role in certain types of NOX-dependent NET formation^57^, and the catalytic activity of Akt is required for both NOX-dependent and -independent NET formation^38^. These previous findings indicate that NET formation can be induced in a variety of ways depending on its triggering stimuli and other contributing factors. Thus, future studies should be directed to focus on elucidating the molecular events required for nsPEF-induced DNA extrusion and histone citrullination, which will lead to a better understanding of nsPEF actions.

Although this study demonstrates that nsPEFs drive the mechanism for NET formation in differentiated HL-60 cells *in vitro*, it is also conceivable that nsPEFs are able to stimulate neutrophils *in vivo*. NET formation in the human body is elicited by pathogens, and various chemical compounds are used for its experimental induction in animal models. Compared to pathogens and chemical compounds, nsPEFs do not leave noxious pathogens or chemicals in the body and might thus serve as a “clean” stimulus for neutrophils *in vivo*. On the other hand, excessive NET formation is known to promote pathogenesis of various diseases for multiple reasons. First, histone proteins in the extracellular space exhibit significant cytotoxicity against host cells in addition to bactericidal activity^58^. Because NETs consist of histone-containing extracellular chromatin fibers, they may cause tissue damage^59^. Secondly, excessive NETs may disturb microcirculation by thrombosis and are thereby potentially detrimental to various organs, particularly highly vascularized ones such as lung and liver^29,60,61^. Lastly, extracellular chromatin DNA and its associated proteins can serve as sources of autoantigens, promoting the onset of autoimmune diseases. From these points of view, nsPEFs could have both positive and negative effects when applied to the human body. Despite the advantageous features of nsPEFs as a clean stimulus for NET formation and as an effective therapeutic modality for cancer, careful considerations are required for the proper application of nsPEFs to the human body in order to avoid adverse outcomes. Thus, the evaluation of nsPEF-induced NET formation in more physiologically relevant settings deserves further investigation.

## Methods

### Antibodies and reagents

An anti-histone H3 antibody was obtained from Cell Signaling Technology (#4499, MA, USA). An antibody against citrullinated histone H3 at R2 was purchased from Abcam (ab176843, Cambridge, UK). A secondary antibody conjugated with horse radish peroxidase (HRP) was obtained from Santa Cruz Biotechnologies (Dallas, TX, USA). The following reagents were used in this study: SYTOX Green and YO-PRO-1 (Thermo Fisher Scientific, MA, USA), MitoRed and Hoechst 33342 (Dojindo Laboratories, Kumamoto, Japan), A23187 and ionomycin (Wako Pure Chemical, Osaka, Japan), MNase (New England BioLabs, MA, USA), and RNase A (Sigma-Aldrich, MO, USA).

### Cell culture

The following cells were obtained from RIKEN (Wako, Japan): HL-60, Jurkat, K562, and THP-1. Cells were grown in RPMI1640 medium (Wako Pure Chemical) supplemented with 10% heat-inactivated fetal bovine serum (FBS, Corning, NY, USA), 100 µg/mL streptomycin, and 100 units/mL penicillin. Cells were cultured under standard conditions at 37 °C in a humidified incubator containing 5% CO_2_. To allow for differentiation into neutrophils, HL-60 cells were collected by brief centrifugation, resuspended in RPMI medium containing 1.3% DMSO at 1 × 10^5^ cells/mL, and cultured for 3 days. Cell numbers were determined using a Z1 Coulter particle counter (Beckman Coulter, CA, USA).

### Generation of nsPEFs

Shots of nsPEFs were generated as described previously^10,11^. Briefly, the electronic system for the generation of nsPEFs (Pulsed Power Modulator MPC3000S) was manufactured by Suematsu Electronics Co., Ltd. (Kumamoto, Japan). Voltage waveforms of the electric pulses were monitored using a P6015A high voltage probe and a DPO4054 digital phosphor oscilloscope (Tektronix, OR, USA). The wave form of nsPEFs under our standard experimental conditions has been described previously^10^. The average pulse width at half maximum was estimated to be approximately 80 ns^10^.

### Exposure of cells to nsPEFs

HL-60 cells were collected by brief centrifugation and suspended in Hepes-buffered saline (HBS; 10 mM Hepes pH 7.4, 140 mM NaCl, 10 mM KCl, 2 mM CaCl_2_, 0.1% glucose, and 0.2 mg/ml bovine serum albumin). The cell suspension (400 µl) was placed in an electroporation cuvette that contained a pair of parallel aluminum electrodes with a 4 mm-gap (BioRad, CA, USA). nsPEFs at 1 Hz were generated as described above and applied to the cell suspension in the cuvette.

### Microscopy

An aliquot of cell suspension exposed to nsPEFs was placed in a glass bottom dish (Matsunami, Osaka, Japan) and incubated at 37 °C. For cell staining, SYTOX Green (2 µM), Hoechst 33342 (1 µg/ml) and MitoRed (0.2 µM) were added to the cell suspension, and microscopy was performed using an FV1200-IX83 laser scanning confocal microscope (Olympus, Tokyo, Japan). The microscope was equipped with a stage top incubator (Tokai Hit, Shizuoka, Japan) that maintained a humidified atmosphere of 5% CO_2_ at 37 °C. Images were analyzed using FLUOVIEW software (Olympus).

### Analysis of nuclear lobulation

Cells were stained with Hoechst 33342 (1 µg/ml) and MitoRed (0.2 µM), and microscopy was performed as described above. Cells exhibiting the invagination of the nuclear rim at one or more sites were scored as nuclear lobulated cells, and at least 200 cells were inspected by microscopy in each experiments. Experiments were repeated 6 times.

### YO-PRO-1 staining

Cell suspension containing 1 µM YO-PRO-1 was exposed to nsPEFs and placed in a glass bottom dish. At 5 min after nsPEF exposure, fluorescent images were acquired using the identical microscopical settings. Fluorescence of individual cells was quantified using FLUOVIEW software (Olympus) and expressed in arbitrary units.

### Gel-electrophoretic analysis of extracellular DNA

To dissociate NETs from their originating cells, cell suspension (1 × 10^6^ cells/ml in HBS) was treated with 0.2 unit/µl MNase at room temperature for 5 min. MNase reaction was stopped by adding EDTA at 10 mM, and cells were pelleted by centrifugation at 200 × *g* for 2 min. The DNA fragments in the supernatant were purified by proteinase K treatment followed by ethanol precipitation. Purified DNA fragments were resolved by agarose gel electrophoresis and subsequently visualized by ethidium bromide staining according to standard procedures.

### Fluorometric measurement of extracellular DNA

For the measurement of extracellular DNA, cell suspension was treated with 0.1 unit/µl MNase and 1 µg/ml RNase A at room temperature for 5 min. The MNase reaction was stopped by adding EDTA at 10 mM, and the cells were removed by centrifugation at 200 × *g* for 2 min. SYTOX Green was added to the supernatant at 2.5 µM, and fluorescence was measured using a 2030 ARVO X multilabel reader (Perkin Elmer, MA, USA). For the measurement of total DNA, cells were suspended in HBS containing 0.5% Triton X-100 and lysed by three cycles of freeze-thaw. Cell lysates were reacted with 0.1 unit/µl MNase and 1 µg/ml RNase A at room temperature for 5 min. EDTA (10 mM) and SYTOX Green (2.5 µM) were added to the lysates, and fluorometric measurement was performed as described above. DNA extrusion was expressed as a ratio of fluorescence for extracellular DNA to that for total DNA. When Ca^2+^-free HBS was used (Fig. 6D), CaCl_2_ solution was added to cell suspension prior to MNase treatment to yield 2 mM Ca^2+^, as MNase requires Ca^2+^ for its catalytic activity.

### Western blotting

Cell suspension (1 × 10^7^ cells/ml in HBS) was exposed to nsPEFs, immediately diluted 5-fold into pre-warmed HBS, and incubated at 37 °C for the appropriate time periods. Cells were collected by centrifugation and then snap-frozen in liquid nitrogen. Cells were lysed in SDS–PAGE loading buffer containing 1% SDS and then sonicated using a microsonicator (Model UR-20P, Tomy Seiko, Tokyo, Japan). Cell lysates were cleared by brief centrifugation and in turn subjected to SDS-polyacrylamide gel electrophoresis followed by western blot analysis as described previously^10^. Antigen–antibody complexes were reacted with an HRP-conjugated secondary antibody and then incubated in Super Signal West Pico reagent (Thermo Fisher Scientific). Chemiluminescence was detected using ChemiDoc XRS Plus analyzer (BioRad).

### RT-PCR

Total RNA was extracted from the cells by the acid guanidinium-phenol-chloroform method^62^ using RNAiso plus (Takara Bio). Total RNA (20–200 ng) was subjected to reverse transcription followed by PCR using OneStep RT-PCR Kit (QIAGEN) with gene-specific primers. PCR products were separated by agarose gel electrophoresis and visualized by staining with ethidium bromide. The primer sequences used in this study were as follows:

CD11b- forward, 5′-CAGAGCGTGGTCCAGCTTCAG-3′;

CD11b- reverse, 5′-CCTTCATCCGCCGAAAGTCAT-3′;

hTERT- forward, 5′-TTTCTGGATTTGCAGGTGAA-3′;

hTERT- reverse, 5′-CAGGAAAAATGTGGGGTTCT-3′;

GAPDH- forward, 5′-ACCACAGTCCATGCCATCAC-3′;

GAPDH- reverse, 5′-TCCACCACCCTGTTGCTGTA-3′;

### Measurement of cell viability

Cell suspension was prepared in RPMI1640 medium supplemented with 10% FBS and antibiotics and exposed to nsPEFs as described above. At 6 h after nsPEF exposure, cell viability was analyzed using a CellTiter-Glo luminescent cell viability assay kit (Promega, WI, USA) according to the manufacturer’s procedures. Luminescence was measured using a 2030 ARVO X multilabel reader (Perkin Elmer).

## Supplementary information

Supplementary Information

## Data Availability

The datasets generated during and/or analyzed during the current study are available from the corresponding author on reasonable request.

## References

[1] Pakhomov AG (2009). Lipid nanopores can form a stable, ion channel-like conduction pathway in cell membrane. Biochem. Biophys. Res. Commun..

[2] Vernier P.Thomas, Sun Yinghua, Marcu Laura, Salemi Sarah, Craft Cheryl M, Gundersen Martin A (2003). Calcium bursts induced by nanosecond electric pulses. Biochemical and Biophysical Research Communications.

[3] White JA, Blackmore PF, Schoenbach KH, Beebe SJ (2004). Stimulation of capacitative calcium entry in HL-60 cells by nanosecond pulsed electric fields. J. Biol. Chem..

[4] Semenov I, Xiao S, Pakhomov AG (2013). Primary pathways of intracellular Ca(2+) mobilization by nanosecond pulsed electric field. Biochim. Biophys. Acta.

[5] Vernier PT, Sun Y, Gundersen MA (2006). Nanoelectropulse-driven membrane perturbation and small molecule permeabilization. BMC Cell Biol..

[6] Muratori C (2017). Activation of the phospholipid scramblase TMEM16F by nanosecond pulsed electric fields (nsPEF) facilitates its diverse cytophysiological effects. J. Biol. Chem..

[7] Morotomi-Yano K, Akiyama H, Yano K (2012). Nanosecond pulsed electric fields activate AMP-activated protein kinase: implications for calcium-mediated activation of cellular signaling. Biochem. Biophys. Res. Commun..

[8] Tolstykh GP (2013). Activation of intracellular phosphoinositide signaling after a single 600 nanosecond electric pulse. Bioelectrochemistry.

[9] Morotomi-Yano K, Uemura Y, Katsuki S, Akiyama H, Yano K (2011). Activation of the JNK pathway by nanosecond pulsed electric fields. Biochem. Biophys. Res. Commun..

[10] Morotomi-Yano K, Akiyama H, Yano K (2011). Nanosecond pulsed electric fields activate MAPK pathways in human cells. Arch. Biochem. Biophys..

[11] Morotomi-Yano K, Oyadomari S, Akiyama H, Yano K (2012). Nanosecond pulsed electric fields act as a novel cellular stress that induces translational suppression accompanied by eIF2alpha phosphorylation and 4E-BP1 dephosphorylation. Exp. Cell Res..

[12] Beebe SJ, Fox PM, Rec LJ, Willis EL, Schoenbach KH (2003). Nanosecond, high-intensity pulsed electric fields induce apoptosis in human cells. FASEB J..

[13] Ren W, Sain NM, Beebe SJ (2012). Nanosecond pulsed electric fields (nsPEFs) activate intrinsic caspase-dependent and caspase-independent cell death in Jurkat cells. Biochem. Biophys. Res. Commun..

[14] Morotomi-Yano K, Akiyama H, Yano K (2013). Nanosecond pulsed electric fields induce poly(ADP-ribose) formation and non-apoptotic cell death in HeLa S3 cells. Biochem. Biophys. Res. Commun..

[15] Morotomi-Yano K, Akiyama H, Yano K (2014). Different involvement of extracellular calcium in two modes of cell death induced by nanosecond pulsed electric fields. Arch. Biochem. Biophys..

[16] Morotomi-Yano K, Yano KI (2017). Calcium-dependent activation of transglutaminase 2 by nanosecond pulsed electric fields. FEBS Open Bio.

[17] Nuccitelli R (2017). Nano-Pulse Stimulation is a physical modality that can trigger immunogenic tumor cell death. J. Immun. Cancer.

[18] Guo S (2018). Nano-pulse stimulation induces potent immune responses, eradicating local breast cancer while reducing distant metastases. Int. J. Cancer.

[19] Schoenbach KH, Beebe SJ, Buescher ES (2001). Intracellular effect of ultrashort electrical pulses. Bioelectromagnetics.

[20] Wang S (2009). Cardiac myocyte excitation by ultrashort high-field pulses. Biophys. J..

[21] Semenov I (2018). Excitation and injury of adult ventricular cardiomyocytes by nano- to millisecond electric shocks. Sci. Rep..

[22] Roth CC (2013). Nanosecond pulsed electric field thresholds for nanopore formation in neural cells. J. Biomedi. Optics.

[23] Pakhomov AG, Semenov I, Casciola M, Xiao S (2017). Neuronal excitation and permeabilization by 200-ns pulsed electric field: An optical membrane potential study with FluoVolt dye. Biochim. Biophys. Acta..

[24] Zhang J (2008). Nanosecond pulse electric field (nanopulse): a novel non-ligand agonist for platelet activation. Arch. Biochem. Biophys..

[25] Vernier PT, Sun Y, Chen MT, Gundersen MA, Craviso GL (2008). Nanosecond electric pulse-induced calcium entry into chromaffin cells. Bioelectrochemistry.

[26] Kolaczkowska E, Kubes P (2013). Neutrophil recruitment and function in health and inflammation. Nat. Rev. Immunology.

[27] Jorgensen I, Rayamajhi M, Miao EA (2017). Programmed cell death as a defence against infection. Nat. Rev. Immunol..

[28] Farrera C, Fadeel B (2013). Macrophage clearance of neutrophil extracellular traps is a silent process. J. Immunol..

[29] Fuchs TA (2010). Extracellular DNA traps promote thrombosis. Proc. Natl. Acad. Sci. USA.

[30] Wen F, White GJ, VanEtten HD, Xiong Z, Hawes MC (2009). Extracellular DNA is required for root tip resistance to fungal infection. Plant Physiol..

[31] Altincicek B, Stotzel S, Wygrecka M, Preissner KT, Vilcinskas A (2008). Host-derived extracellular nucleic acids enhance innate immune responses, induce coagulation, and prolong survival upon infection in insects. J. Immunol..

[32] Zhang X, Zhuchenko O, Kuspa A, Soldati T (2016). Social amoebae trap and kill bacteria by casting DNA nets. Nat. Commun..

[33] Wang Y (2009). Histone hypercitrullination mediates chromatin decondensation and neutrophil extracellular trap formation. J. Cell Biol..

[34] Knuckley B (2010). Substrate specificity and kinetic studies of PADs 1, 3, and 4 identify potent and selective inhibitors of protein arginine deiminase 3. Biochemistry.

[35] Collins SJ, Ruscetti FW, Gallagher RE, Gallo RC (1978). Terminal differentiation of human promyelocytic leukemia cells induced by dimethyl sulfoxide and other polar compounds. Proc. Natl. Acad. Sci. USA.

[36] Rosmarin AG (1989). Differential expression of CD11b/CD18 (Mo1) and myeloperoxidase genes during myeloid differentiation. Blood.

[37] Xu D, Gruber A, Bjorkholm M, Peterson C, Pisa P (1999). Suppression of telomerase reverse transcriptase (hTERT) expression in differentiated HL-60 cells: regulatory mechanisms. Brit. J. Cancer.

[38] Douda DN, Khan MA, Grasemann H, Palaniyar N (2015). SK3 channel and mitochondrial ROS mediate NADPH oxidase-independent NETosis induced by calcium influx. Proc. Natl. Acad. Sci. USA.

[39] Pakhomova ON, Gregory B, Semenov I, Pakhomov AG (2014). Calcium-mediated pore expansion and cell death following nanoelectroporation. Biochim. Biophys. Acta.

[40] Steelman ZA, Tolstykh GP, Beier HT, Ibey BL (2016). Cellular response to high pulse repetition rate nanosecond pulses varies with fluorescent marker identity. Biochem. Biophys. Res. Commun..

[41] Stacey M (2003). Differential effects in cells exposed to ultra-short, high intensity electric fields: cell survival, DNA damage, and cell cycle analysis. Mut. Res..

[42] Garon EB (2007). *In vitro* and *in vivo* evaluation and a case report of intense nanosecond pulsed electric field as a local therapy for human malignancies. Int. J. Cancer.

[43] Ibey BL (2010). Selective cytotoxicity of intense nanosecond-duration electric pulses in mammalian cells. Biochim. Biophys. Acta.

[44] Ibey BL (2011). Dose-dependent thresholds of 10-ns electric pulse induced plasma membrane disruption and cytotoxicity in multiple cell lines. PLoS One.

[45] Yang W (2011). Differential sensitivities of malignant and normal skin cells to nanosecond pulsed electric fields. Technol. Cancer Res. Treatment.

[46] Yin D (2012). Cutaneous papilloma and squamous cell carcinoma therapy utilizing nanosecond pulsed electric fields (nsPEF). PLoS One.

[47] Gianulis EC (2017). Selective susceptibility to nanosecond pulsed electric field (nsPEF) across different human cell types. Cell. Mol. Life Sci..

[48] Munoz LE, Kaplan MJ, Radic M, Herrmann M (2017). Editorial: NETosis 2: The Excitement Continues. Front. Immunol..

[49] Li Y, Cao X, Liu Y, Zhao Y, Herrmann M (2018). Neutrophil extracellular traps formation and aggregation orchestrate induction and resolution of sterile crystal-mediated inflammation. Front. Immunol..

[50] Kenny, E. F. *et al*. Diverse stimuli engage different neutrophil extracellular trap pathways. eLife **6**, 10.7554/eLife.24437 (2017). 10.7554/eLife.24437 PMC5496738 28574339

[51] van der Linden M, Westerlaken GHA, van der Vlist M, van Montfrans J, Meyaard L (2017). Differential signalling and kinetics of neutrophil extracellular trap release revealed by quantitative live imaging. Sci. Rep..

[52] de Bont CM, Koopman WJH, Boelens WC, Pruijn GJM (2018). Stimulus-dependent chromatin dynamics, citrullination, calcium signalling and ROS production during NET formation. Biochim. Biophys. Acta.

[53] Arai Y (2014). Uric acid induces NADPH oxidase-independent neutrophil extracellular trap formation. Biochem. Biophys. Res. Commun..

[54] Pakhomova ON (2012). Oxidative effects of nanosecond pulsed electric field exposure in cells and cell-free media. Arch. Biochem. Biophys..

[55] Nuccitelli R, Lui K, Kreis M, Athos B, Nuccitelli P (2013). Nanosecond pulsed electric field stimulation of reactive oxygen species in human pancreatic cancer cells is Ca(2+)-dependent. Biochem. Biophys. Res. Commun..

[56] Hakkim A (2011). Activation of the Raf-MEK-ERK pathway is required for neutrophil extracellular trap formation. Nat. Chem. Biol..

[57] Khan MA (2017). JNK Activation Turns on LPS- and Gram-Negative Bacteria-Induced NADPH Oxidase-Dependent Suicidal NETosis. Sci. Rep..

[58] Xu J (2009). Extracellular histones are major mediators of death in sepsis. Nat. Med..

[59] Saffarzadeh M (2012). Neutrophil extracellular traps directly induce epithelial and endothelial cell death: a predominant role of histones. PLoS One.

[60] Jorch SK, Kubes P (2017). An emerging role for neutrophil extracellular traps in noninfectious disease. Nat. Med..

[61] Papayannopoulos V (2018). Neutrophil extracellular traps in immunity and disease. Nat. Rev. Immunol..

[62] Nicolaides NC, Stoeckert CJ (1990). A simple, efficient method for the separate isolation of RNA and DNA from the same cells. BioTechniques.

