# Supplementary material for: Nanosecond pulsed electric fields induce extracellular release of chromosomal DNA and histone citrullination in neutrophil-differentiated HL-60 cells
Source: Sci Rep. 2019 Jun 11;9:8451. doi: 10.1038/s41598-019-44817-9 (PMC6559984; doi:10.1038/s41598-019-44817-9)
Supplement: Supplementary file 1 — Supplementary Information [file 41598_2019_44817_MOESM1_ESM.pdf]

Supplementary Information

Nanosecond pulsed electric fields induce extracellular release of chromosomal DNA and histone citrullination in neutrophil-differentiated HL-60 cells.

Tsubasa Koga[1], Keiko Morotomi-Yano[2], Takashi Sakugawa[1, 2], Hisato Saitoh[1, 2, 3], Ken-ichi Yano[1, 2, *]

[1]Graduate School of Science and Technology, Kumamoto University, Kumamoto 860-8555, Japan

[2]Institute of Pulsed Power Science, Kumamoto University, Kumamoto 860-8555, Japan

[3]Faculty of Advanced Science and Technology (FAST), Kumamoto University, Kumamoto 860-8555, Japan

*Correspondence should be addressed to K. Y. (e-mail: yanoken@kumamoto-u.ac.jp)

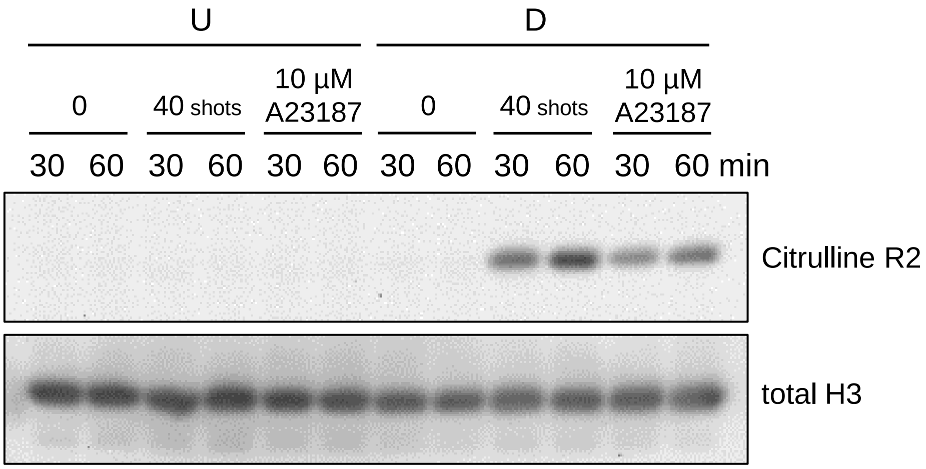

Original Images of Western Blots

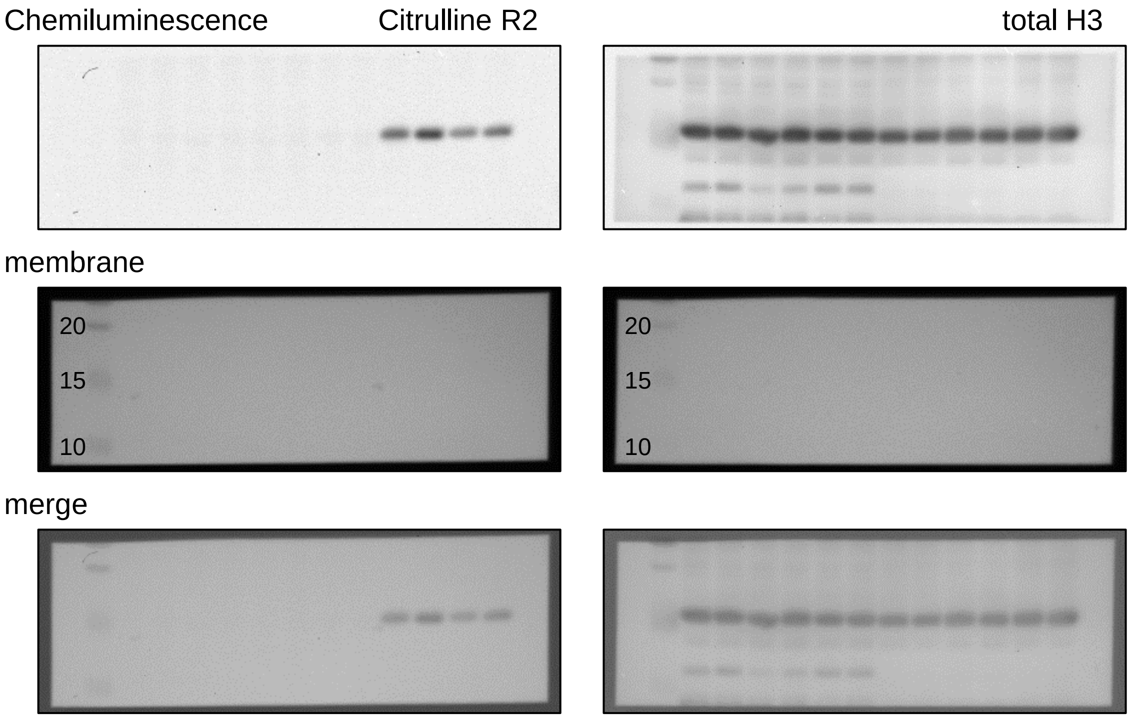

Supplementary Figure S1. Western blot analysis of A23187-induced histone citrullination and original images of the blots.

Differentiated (D) and undifferentiated (U) HL-60 cells were treated with 40 shots of 20 kV/cm nsPEFs or 10 µM A23187. Cells were collected after the indicated time periods and subjected to Western blot analysis of citrullinated histone H3.

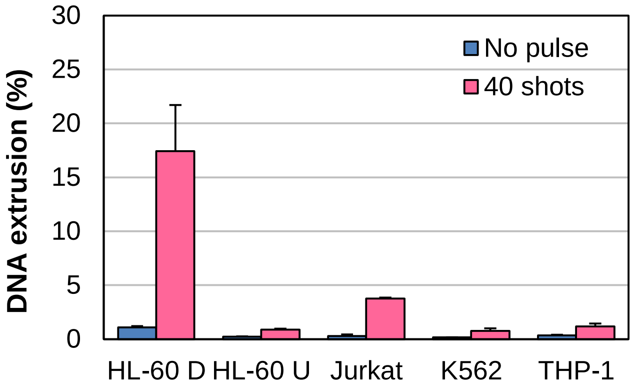

Supplementary Figure S2. Effects of 40 shots of 20 kV/cm nsPEFs on DNA extrusion in HL-60 and other cell lines.

Differentiated (D) and undifferentiated (U) HL-60, Jurkat, K562, and THP-1 cells were exposed to 40 shots of 20 kV/cm nsPEFs. After 1 hr incubation, fluorometric analysis of DNA extrusion was performed. Average values with SD were calculated from 5 independent experiments

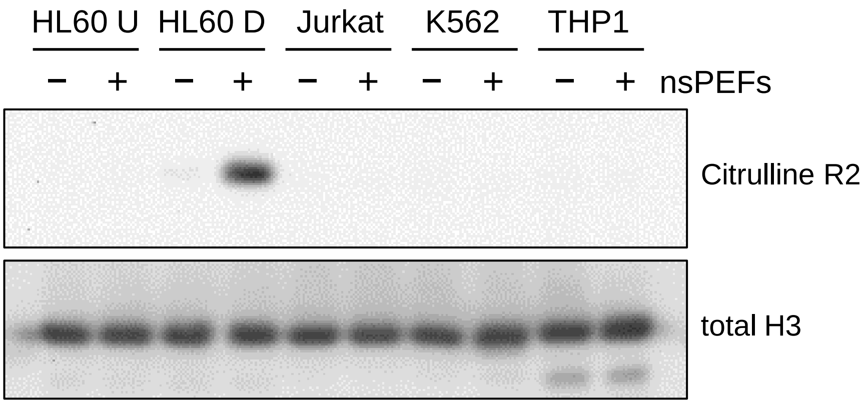

Original Images of Western Blots

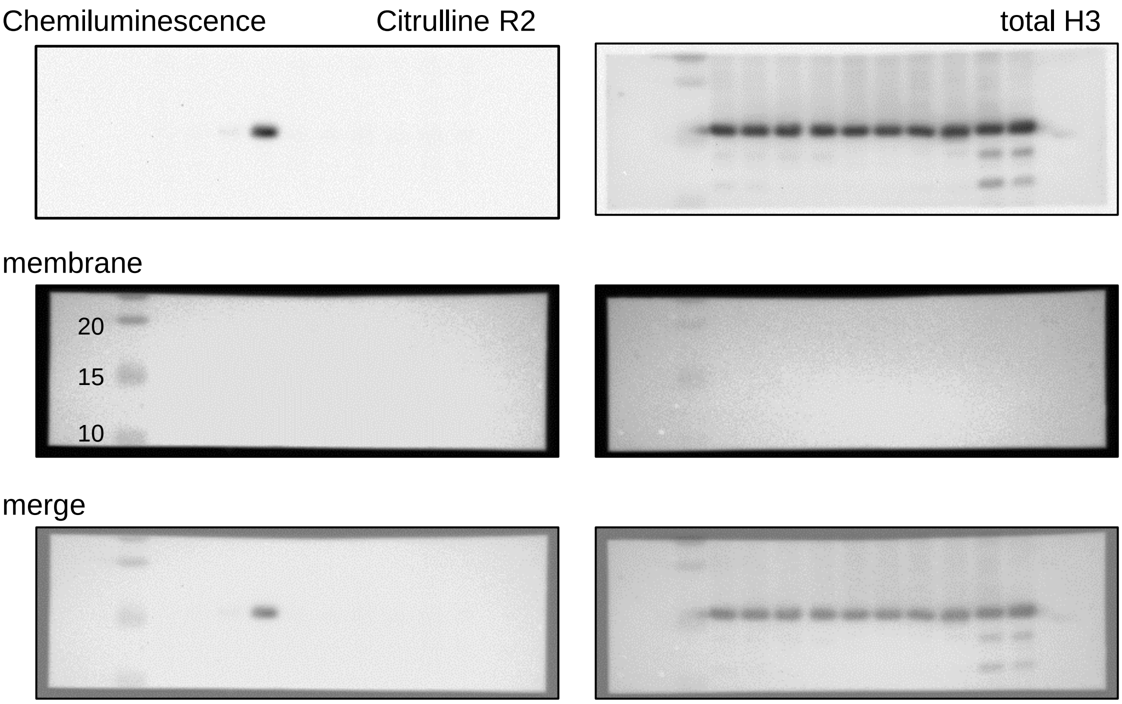

Supplementary Figure S3. Western blot analysis of histone citrullination in HL-60 and other cell lines and original images of the blots.

Cells were treated with (+) and without (-) 40 shots of 20 kV/cm nsPEFs and incubated at 37°C for 30 min.

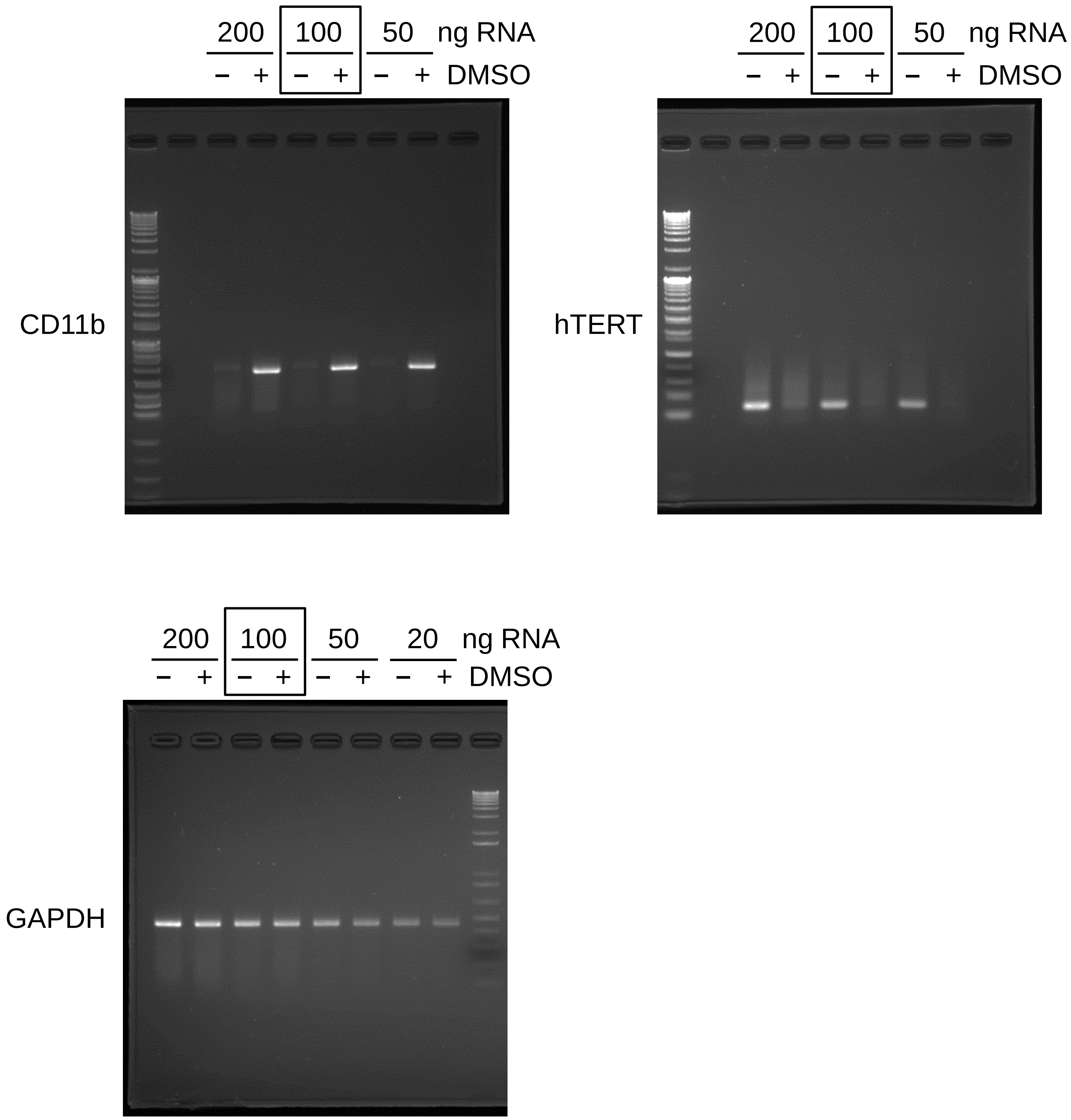

Supplementary Figure S4. Uncropped images used in Fig. 1E.

Different amounts of total RNA were used for RT-PCR in parallel. Results of RT-PCR analysis using 100 ng total RNA were shown in Fig. 1E.

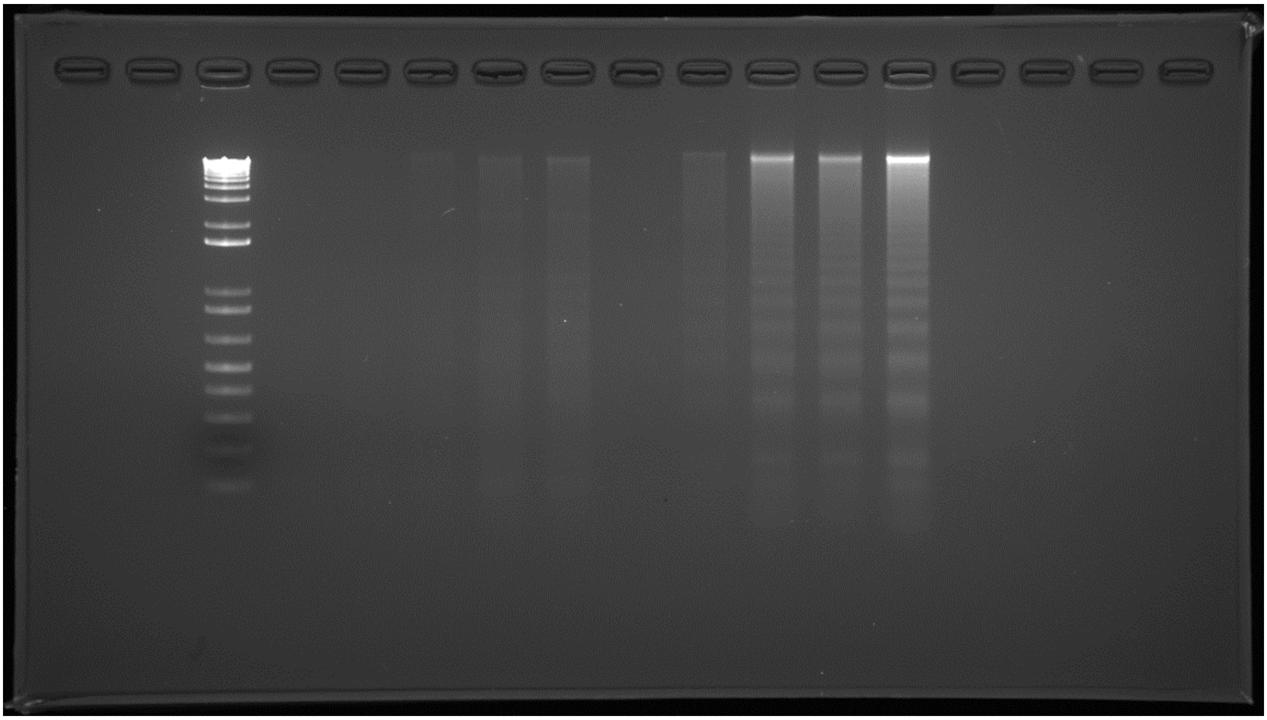

Supplementary Figure S5. Uncropped image used in Fig. 3B.

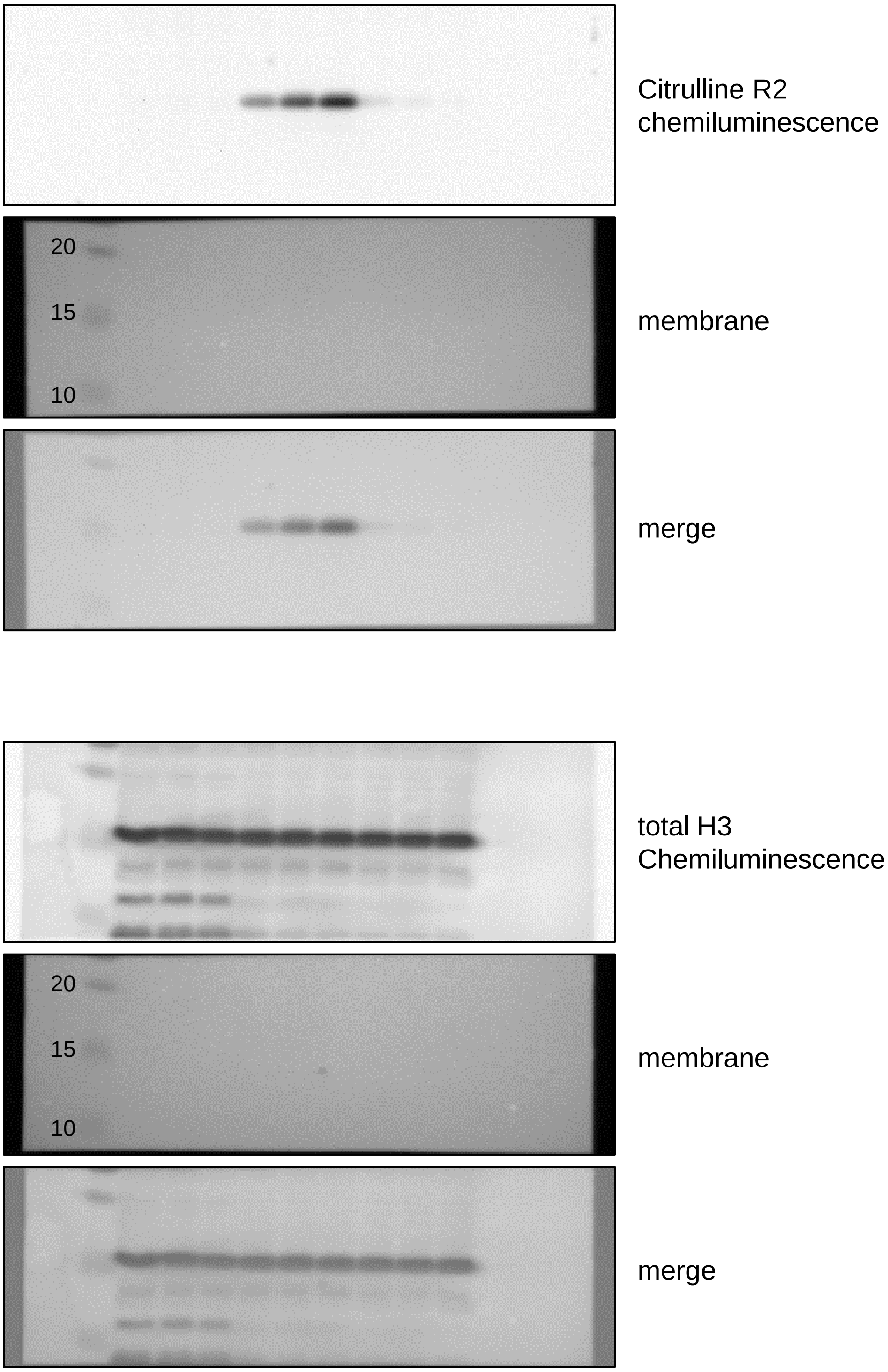

Supplementary Figure S6. Original western blot images used in Fig. 5B. Images for chemiluminescence and a blotted membrane with molecular weight markers are shown. Numbers indicate molecular weights (kDa).

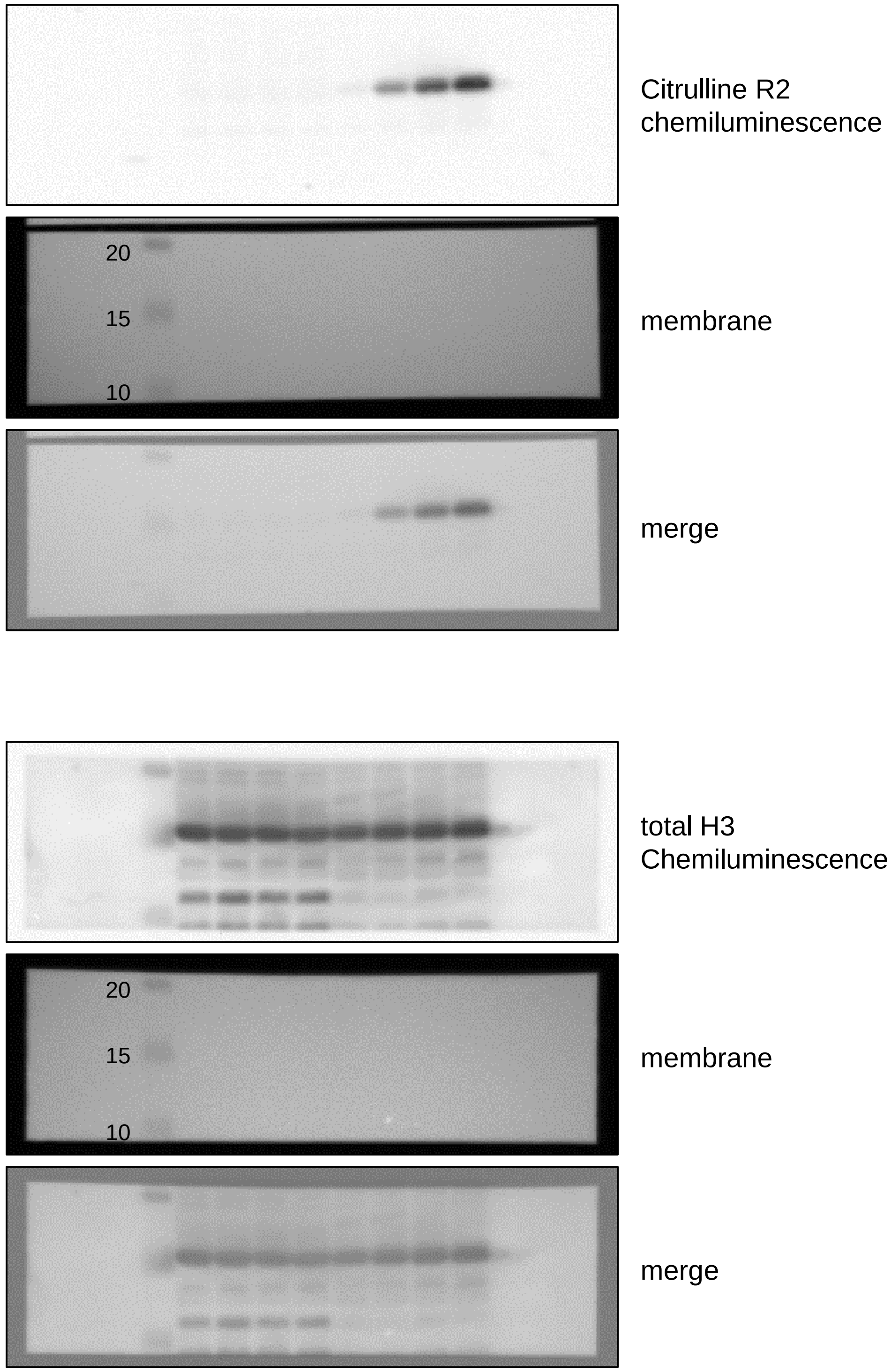

Supplementary Figure S7. Original western blot images used in Fig. 5C. Images for chemiluminescence and a blotted membrane with molecular weight markers are shown. Numbers indicate molecular weights (kDa).

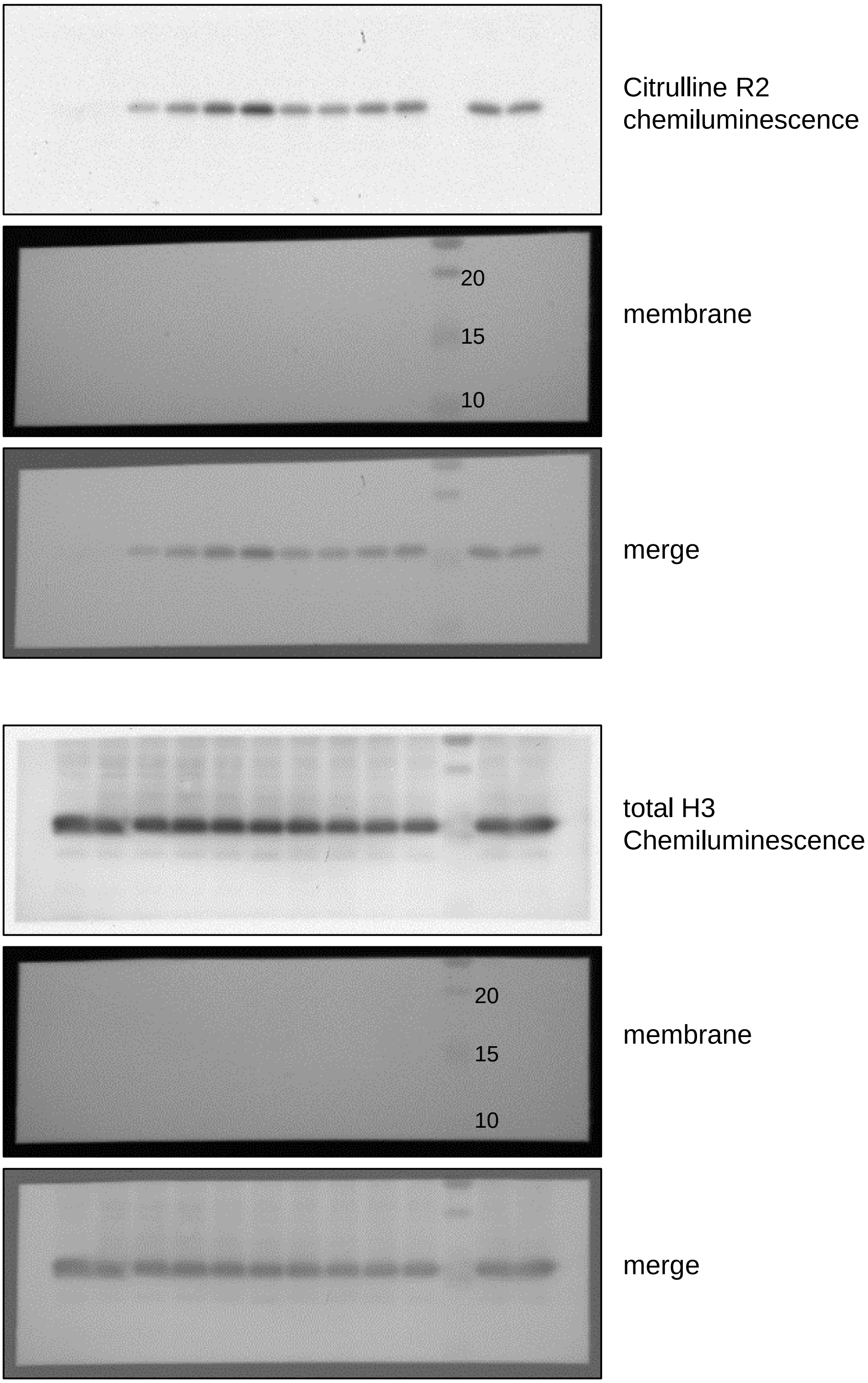

Supplementary Figure S8. Original western blot images used in Fig. 5D. Images for chemiluminescence and a blotted membrane with molecular weight markers are shown. Numbers indicate molecular weights (kDa).

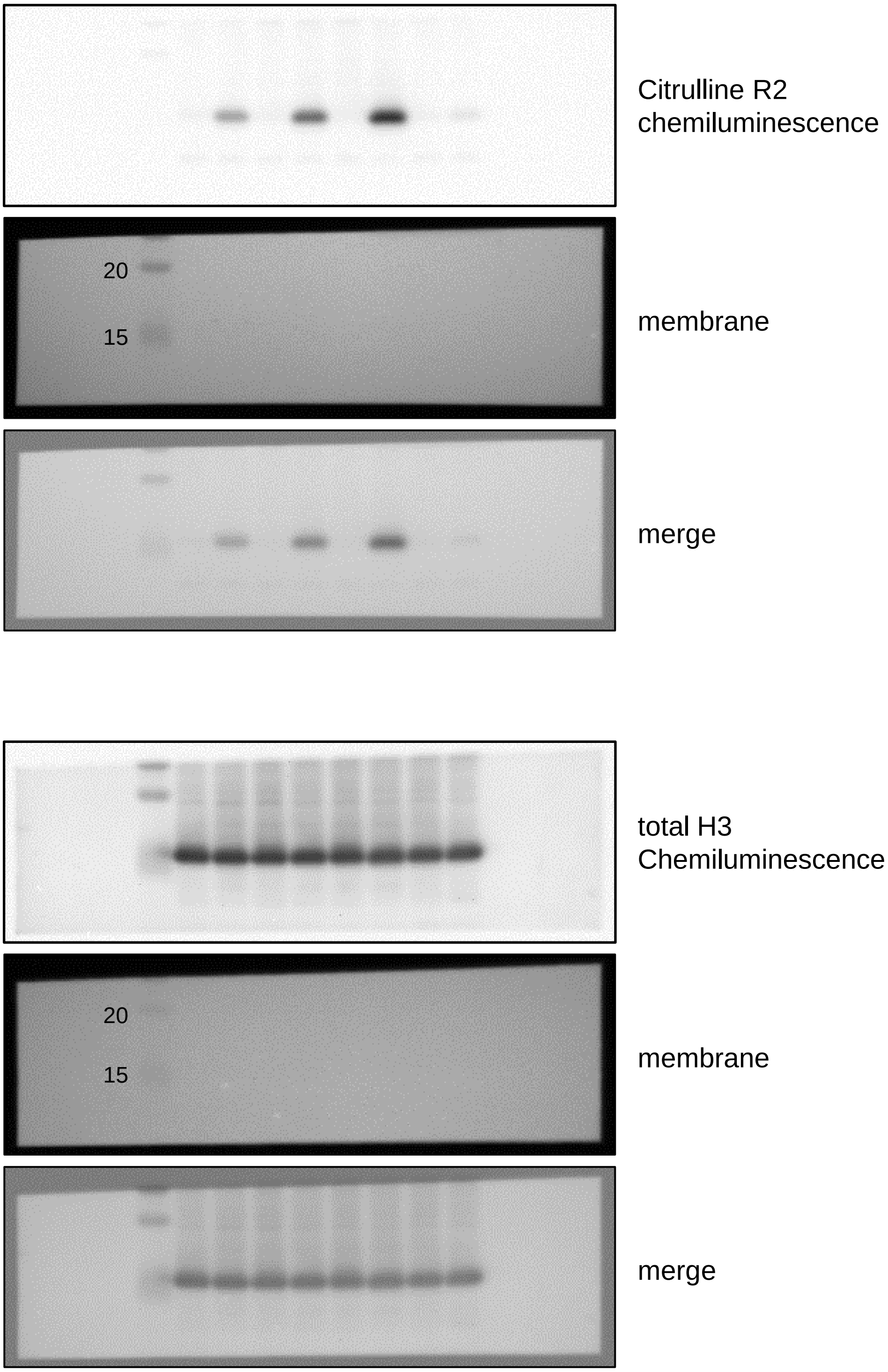

Supplementary Figure S9. Original western blot images used in Fig. 6E. Images for chemiluminescence and a blotted membrane with molecular weight markers are shown. Numbers indicate molecular weights (kDa).

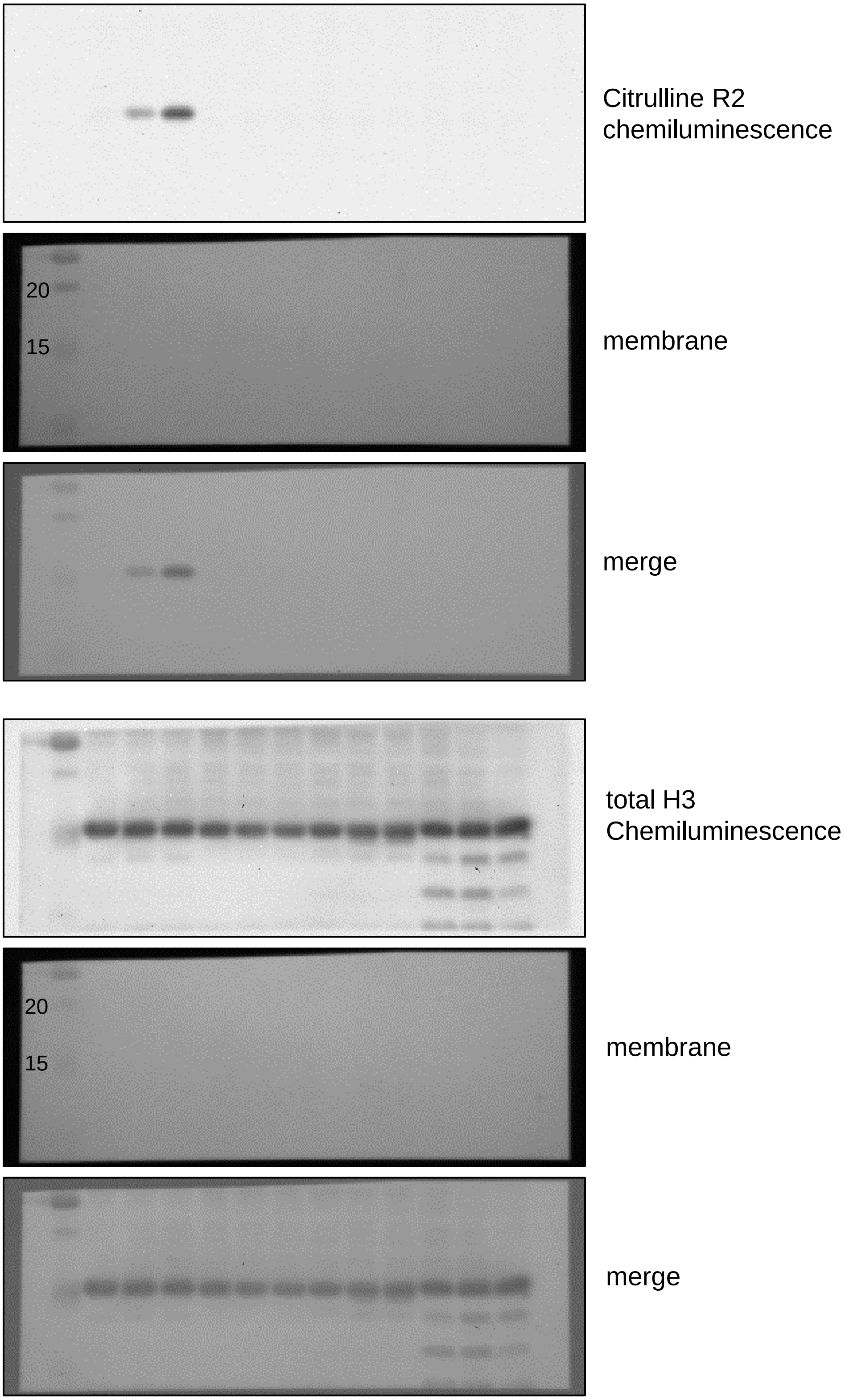

Supplementary Figure S10. Original western blot images used in Fig. 7C.

Images for chemiluminescence and a blotted membrane with molecular weight markers are shown. Numbers indicate molecular weights (kDa).
